# Exosomal circTUBGCP4 promotes vascular endothelial cell tipping and colorectal cancer metastasis by activating Akt signaling pathway

**DOI:** 10.1186/s13046-023-02619-y

**Published:** 2023-02-15

**Authors:** Chen Chen, Yang Liu, Lin Liu, Chaohua Si, Yanxin Xu, Xiaoke Wu, Chengzeng Wang, Zhenqiang Sun, Qiaozhen Kang

**Affiliations:** 1 School of Life Science, Zhengzhou University, Zhengzhou, 450001 Henan China; 2 Department of Colorectal Surgery, The First Affiliated Hospital of Zhengzhou University, #1 Jianshe East Road, Zhengzhou, 450052 Henan China; 3 Henan Institute of Interconnected Intelligent Health Management, The First Affiliated Hospital of Zhengzhou University, Zhengzhou, Henan 450052 China; 4 Department of Radiotherapy, Affiliated Cancer Hospital of Zhengzhou University, Henan Cancer Hospital, Zhengzhou, Henan 450008 China; 5 Department of Ultrasound, The First Affiliated Hospital of Zhengzhou University, Zhengzhou, 450052 Henan China; 6 Academy of Medical Sciences, Zhengzhou University, Zhengzhou, 450052 Henan China; 7 Department of Neurology, The First Affiliated Hospital of Zhengzhou University, Zhengzhou, 450052 Henan China

**Keywords:** Exosome, CircRNA, Tip cells formation, Angiogenesis, Tumor metastasis

## Abstract

**Background:**

Exosome is crucial mediator and play an important role in tumor angiogenesis. Tip cell formation is a prerequisite for persistent tumor angiogenesis which causes tumor metastasis. However, the functions and underlying mechanisms of tumor cell-derived exosomes in angiogenesis and tip cell formation remain less understood.

**Methods:**

Exosomes derived from serum of colorectal cancer (CRC) patients with metastasis/non-metastasis and CRC cells were isolated by ultracentrifugation. CircRNAs in these exosomes were analyzed by circRNA microarray. Then, exosomal circTUBGCP4 was identified and verified by quantitative real-time PCR (qRT–PCR) and in situ hybridization (ISH). Loss- and gain-of-function assays were performed to explore the effect of exosomal circTUBGCP4 on vascular endothelial cell tipping and colorectal cancer metastasis in vitro and in vivo. Mechanically, bioinformatics analysis, biotin-labeled circTUBGCP4/ miR-146b-3p RNA pulldown, RNA immunoprecipitation (RIP), and luciferase reporter assay were used to confirm the interaction among circTUBGCP4, miR-146b-3p, and PDK2.

**Results:**

Here, we showed that exosomes derived from CRC cells enhanced vascular endothelial cell migration and tube formation via inducing filopodia formation and endothelial cell tipping. We further screened the upregulated circTUBGCP4 in serum of CRC patients with metastasis compared to non-metastasis. Silencing circTUBGCP4 expression in CRC cell-derived exosomes (CRC-CDEs) inhibited endothelial cell migration, tube formation, tip cell formation, and CRC metastasis. Overexpression of circTUBGCP4 had opposite results in vitro and in vivo. Mechanically, circTUBGCP4 upregulated PDK2 to activate Akt signaling pathway by sponging miR-146b-3p. Moreover, we found that miR-146b-3p could be a key regulator for vascular endothelial cell dysfunction. Exosomal circTUBGCP4 promoted tip cell formation and activated the Akt signaling pathway by inhibiting miR-146b-3p.

**Conclusions:**

Our results suggest that colorectal cancer cells generate exosomal circTUBGCP4, which causes vascular endothelial cell tipping to promote angiogenesis and tumor metastasis by activating Akt signaling pathway.

**Supplementary Information:**

The online version contains supplementary material available at 10.1186/s13046-023-02619-y.

## Background

CRC is still one of the most morbidity and mortality cancers in China [1]. Metastasis is the leading cause of high mortality in CRC patients. Effective control of tumor metastasis is the main goal that needs to be explored in the future. Neovascularization and angiogenesis are homeostatic processes for transporting nutrients in physiological systems. However, homeostasis is disrupted in the tumor tissue. Intratumoral vascular overgrowth forms a disorder and unstable vascular network that accelerates tumor growth, invasion, and metastasis. In the 1970s, Folkman provide an important hypothesis that anti-angiogenesis could be a strategy to interdict tumor progression [2]. At present, anti-angiogenesis agents, such as bevacizumab targeting VEGF, were used in the targeted therapy of metastatic CRC. However, there are still some metastatic CRC patients who do not benefit from it. Therefore, further understanding of tumor angiogenesis still needs to be explored.

Exosomes, which are a class of vesicles about 30 to 150 nm in diameter, have attracted attention as a new type of delivery carrier transporting proteins and nucleic acid, such as non-coding RNA [3]. Recently, increase studies reported exosomes can be an excellent medium of cell-to-cell communication in changing tumor microenvironment and leading to tumor metastasis [4, 5]. Moreover, non-coding RNA in exosomes from cancer cells or others, such as M2 macrophages, play critical roles in angiogenesis and tumor metastasis [6, 7]. CircRNAs are a class of structurally stable non-coding RNAs that participate in multiple processes in tumorigenesis, development, and metastasis. Therefore, circRNAs have great potential as molecular targets for tumor therapy. It has been reported that circRNA is enriched and stable in exosomes and it could be a promising biomarker for cancer diagnosis [8]. However, the role of exosomal circRNA in tumor angiogenesis and metastasis is still unknown.

In this study, we reported that colorectal cancer cell-derived exosomes (CRC-CDEs) enhanced endothelial cell migration and tube formation. A new finding showed that CRC-CDEs induced filopodia formation leading to endothelial cell tipping. Moreover, we deeply explored the circRNA expression profile in serum of CRC patients with metastasis or non-metastasis. We found a new exosomal circRNA, circTUBGCP4, that promoted the dysfunction of vascular endothelial cells and tumor metastasis. Mechanically, we identified that circTUBGCP4 can sponge miR-146b-3p to activate Akt signaling pathway. Moreover, we proved that the functional role of exosomal circTUBGCP4 was dependent on miR-146b-3p, which was a key regulator to inhibit the dysfunction of vascular endothelial cells. These findings may provide a new way of anti-angiogenesis therapy for CRC metastasis.

## Methods

### Patient tissue specimens and cell lines

Eight samples of serum exosomes from CRC patients with metastasis and non-metastasis were obtained from the First Affiliated Hospital of Zhengzhou University. Clinicopathological data on age, gender, tumor grade, and tumor-lymph node-metastasis (TNM) stage were collected. All patients signed informed consent forms, and the protocols were approved by the Ethics Committee of The First Affiliated Hospital of Zhengzhou University. HCT116 cells were obtained from the Shanghai Cell Bank of Chinese Academy of Sciences (Shanghai, China). SW480 cells and Human embryonic kidney 293 T cells (293 T cells) were generous gifts from the Biotherapy Center of The First Affiliated Hospital of Zhengzhou University. The immortal human umbilical vein endothelial cells (HUVECs) were a generous gift from the Key Laboratory of Cardiac Injury and Repair of Henan Province [9]. HCT116 cells and SW480 cells were cultured in high-glucose DMEM (Gibco, Carlsbad, CA, USA), and HUVECs and 293 T cells were cultured in RPMI 1640 (Gibco, Carlsbad, CA, USA) supplemented with 10% fetal bovine serum (Clark Bioscience, Richmond, VA, USA) at 37 °C and 5% CO_2_.

### CircRNA microarray

The serum exosome and HCT116 exosome were isolated by ultracentrifugation. Exosome was lysed to obtain total RNA, which was then treated with RNase R. The enriched circular RNA was amplified and transcribed into fluorescent cRNA utilizing random primer according to Arraystar Super RNA Labeling protocol (Arraystar Inc., Rockville, MD, USA). Then, the labeled cRNAs were hybridized onto the Arraystar Human circRNA Arrays V2 (8 × 15 K, Arraystar) at 65 °C for 17 h in an Agilent Hybridization Oven. After washing, slides were scanned with the Agilent Scanner G2505C. Raw Data was extracted using Agilent Feature Extraction software. A series of data processing including quantile normalization was performed using the R software limma package. GeneSpring software was used to flag “P” or “M” for further differential analyses. The differential condition was set to fold change > 1.5 or fold change < -1.5 and *p* < 0.05.

### Exosomes isolation, characterization, and label

Firstly, the exosome-free fetal bovine serum (Exo-free FBS) was prepared by ultracentrifugation at 120,000 × *g* overnight at 4 °C before use. HCT116 cells and SW480 cells were cultured in a DMEM medium with 10% Exo-free FBS. After 48 h culture, the conditioned media were collected and centrifuged at 500 × *g* for 10 min at 4 °C, then 16,800 × *g* for 30 min at 4 °C. The supernatants were filtrated by a 0.22 μm filter (Millipore, Burlington, MA, USA), followed by 120,000 × g for 70 min at 4 °C. The exosomes were washed with phosphate-buffered saline (PBS). Then, the nanoparticle tracking analysis (NTA) of the exosome was measured by Nicomp Z3000 (Particle Sizing System, Santa Barbara, CA, USA). The exosomes were observed with the transmission electron microscope (TEM) using JEM1400-80 kV (JEOL, Tokyo, Japan). The exosome was labeled by PKH67 dye (Sigma, St. Louis, MO, USA) and then collected by ultracentrifugation. The PKH67-labeled exosomes were co-cultured with HUVECs at 3 h. Then, the HUVECs were fixed and taken photos by confocal laser scanning microscopy (Zeiss, Jena, Germany).

### Transient transfection of microRNA (miRNA) and plasmids

The circTUBGCP4 overexpression vector was constructed using pcDNA3.1-circRNA (Hanbio Biotechnology, Wuhan, China). miR-146b-3p mimic and NC mimic were synthesized by RiboBio (Guangzhou, China). This plasmid contains two repeated sequences named 5’circFrame and 3circFrame, which promote circRNA formation through reverse complementation [10]. Following the manufacturer’s instructions, Lipofectamine 3000 (Invitrogen, Thermo Fisher Scientific, Carlsbad, CA, USA) was used for transient transfection of miRNA mimic. Hieff Trans™ Liposomal Transfection Reagent (Yeasen Biotechnology, Shanghai, China) was used for transient transfection by circTUBGCP4 overexpression vector.

### Construction of stable cell lines

The lentiviral circTUBGCP4 overexpression vector was constructed using pHBLV-CMV-circ (Hanbio Biotechnology, Wuhan, China). Sh-circTUBGCP4-01 and sh-circTUBGCP4-02 were designed and synthesized by Genepharm (Genepharm, Shanghai, China) (Table S1). The lentiviral vectors /pSPAX2 /pMD2G were transfected into 293 T cells. After 48 h and 72 h, the cell supernatant containing virus was collected and concentrated for subsequent stable transfection into HCT116 cells and SW480 cells using Hieff Trans™ Liposomal Transfection Reagent (Yeasen Biotechnology, Shanghai, China). Then, the cancer cell lines were cultured using puromycin (2 μg/mL) to obtain stable cell lines.

### RNA isolation, reverse transcription, and qRT-PCR

Total RNA was isolated using RNAiso Plus reagent (Takala, Dalian, China) and was assured by NanoDrop 2000 (Thermo Fisher Scientific, Carlsbad, CA, USA). Then, 1ug RNA was reverse transcribed to cDNA using Evo M-MLV RT Kit (Accurate biology, Shanghai, China). MiRNA was reverse transcribed using miRNA 1st strand cDNA synthesis kit (Accurate biology, Shanghai, China). Quantification of circRNA, mRNA, and miRNA was performed using qPCR SYBR Green Master Mix (Shanghai, China) according to the manufacturer’s instructions. All data were analyzed and normalized to GAPDH. All primers are listed in Table S2.

### Actinomycin D assay

HCT116 cells were cultured with 100 ng/ml actinomycin D (Merck, Darmstadt, Germany) at 0 h, 4 h, 8 h, 12 h, and 24 h. Then, the cells were lysed for total RNA extraction. The qRT-PCR was performed to analyze the stability of circTUBGCP4 and TUBGCP4.

### RNA in-situ hybridization (ISH)

The CRC tissue slices and paired adjacent normal tissue slices were dewaxed and digested using Protease K. Then, these slices were added H_2_O_2_ for 30 min for blocking, then washed three times with PBS. After being incubated with a prehybridization solution, these slices subsequently hybridized with the circTUBGCP4 probe (Servicebio, Wuhan, China) (Table S3). Then, these slices were visualized using DAB. The H-score was analyzed using Aipathwell (Servicebio, Wuhan, China).

### Western blot analysis, immunofluorescence (IF), and immunohistochemistry (IHC)

Protein in HUVECs was extracted with RIPA buffer containing PMSF (Solarbio, Beijing, China). The supernatant of the lysis and exosome derived from HCT116 and SW480 was quantified with a BCA kit. Then, more details are provided below [11]. The following antibodies were used: anti-CD9 (Abcam, ab263019); anti-CD63 (Abcam, ab134045); anti-TSG101 (Abcam, ab125011); anti-CD34 (Proteintech, 14,486–1-AP); anti-Integrin β1 (Proteintech, 12,594–1-AP); anti-VEGFA (Proteintech,19,003–1-AP); anti-PDK2 (Abcepta, AP7039b); anti-Akt (Abcam, ab179463); anti-p-Akt (Ser473); anti-GAPDH (Proteintech, 60,004–1-Ig). IF and IHC was performed as previously described [11]. IF was performed using anti-CD34 (Proteintech, 14,486–1-AP). Then, the image of IF was obtained using the LSM880 confocal microscope system (Zeiss, Jena, Germany). IHC was performed using anti-CD31 (Proteintech, 11,265–1-AP) and anti-CD34 (Proteintech, 14,486–1-AP). The images of IHC were acquired using a fluorescence microscope system (Olympus, Tokyo, Japan).

### Transwell migration assay, wound healing assay, tube formation assays, and actin-tracker assays

HUVECs were treated with exosome or overexpressed circTUBGCP4 vector. After 48 h cell culture, the modified HUVECs were used for the following assay. For transwell migration ~ 5 × 10^4^ modified HUVECs were seeded in upper chambers with 8-μm pore membranes of 24-well plates (Corning, NY, USA). The RPMI 1640 with 20% FBS was added to lower chambers to induce cell migration. Then, the migrated cells were harvested after 10 h and stained with Giemsa (Solarbio, Beijing, China). The images of the migration cell were captured in four fields using an optical microscope. The migration area (%) was determined by measuring wound healing percentage (%). For wound healing assay, ~ 1 × 10^5^ modified HUVECs were seeded in 12-well plates. After growing to 90%, HUVECs were scratched into a cross-shaped wound using a 10 μl plastic pipette tip. Then, the wound healing image was acquired using an optical microscope. The tube formation ability was determined by measuring the number of tubes. For tube formation assays, 50 μl Growth Factor Reduced Matrigel (Corning, NY, NYC, USA) was added to 96-well plates for 30 min at 37 °C. Then, ~ 2 × 10^4^ modified HUVECs were seeded in 96-well plates embedded GFR-Matrigel. Then, tube formation was found at 4 h or 6 h using a microscope. The tube formation ability was determined by measuring the number of tubes using the Angiogenesis Analyzer. All data processing was counted by Image J. For the actin-tracker assay, the F-actin was marked by Actin-Tracker Red-Rhodamine (Beyotime, Shanghai, China) according to the manufacturer’s instructions, then the image was obtained using the LSM880 confocal microscope system (Zeiss, Jena, Germany). To show the filopodial extensions more clearly, yellow dots were used to mark the ends of the filopodial extensions (red).

### RNA pull-down assay and RNA immunoprecipitation (RIP) assay

The biotin-labeled circTUBGCP4 probe with its NC probe and miR-146b-3p probe with its NC probe were designed and synthesized by Genepharm (Shanghai, China). The sequence of biotin probe was shown in Table S3. Then, the RNA pull-down assay was performed by the RNA pull-down kit (BersinBio, Guangzhou, China) according to the manufacturer’s instructions. The RIP assay was performed with Magna RIP RNA-Binding Protein Immunoprecipitation Kit (Millipore, MA, USA) according to the manufacturer’s instructions. Magnetic beads were incubated with 5 μg of anti-AGO2 (Cell Signaling Technology, 2897) for 30 min at room temperature. More details are provided below [11]. Then, the pulled-down RNA was reverse transcribed using the Revert Aid First Strand cDNA Synthesis Kit (Thermo Fisher Scientific, Carlsbad, CA, USA). The interactions between circTUBGCP4 and miR-146b-3p were assessed by qRT-PCR.

### Luciferase activity assays

The sequence of circTUBGCP4 and its mutant versions according to miR-146b-3p binding sites were amplified and then inserted into luciferase reporter vector psiCHECK2 (Hanbio Biotechnology, Wuhan, China). This assay is usually performed with 293 T cells mainly due to its high efficiency of transfections. Then, co-transfections of circTUBGCP4-wt and circTUBGCP4-mut plasmids with miR-146b-3p mimic and its NC mimic into the 293 T cells were completed using Lipofectamine 3000 (Invitrogen, Thermo Fisher Scientific, Carlsbad, CA, USA). After 48 h transfection, the cell was collected to detect luciferase activity using the Dual-Luciferase Reporter Assay kit (Beyotime, Shanghai, China).

### Animal models

Four-week-old male athymic BALB/c nude mice were purchased from Vital River Laboratory (Beijing, China). All protocols for animal studies were reviewed and approved by the Institutional Animal Care and Use Committee of Zhengzhou University. The stable cell lines of HCT116-LV-NC and HCT116-LV-CircTUBGCP4 (2 × 10^6^ in 100 µL of PBS) were injected via the tail vein. After 30 days and 40 days, the mice were injected intraperitoneally 100 μl D-luciferin potassium salt (Yeasen, Shanghai, China). Then In vivo imaging was acquired with the IVIS Spectrum (PerkinElmer, Waltham, Massachusetts, USA). After 40 days, mice were sacrificed and lungs were removed following HE staining and IHC.

### Statistical analysis

All data were analyzed using GraphPad Prism 9.0 (GraphPad, San Diego, CA, USA) and expressed as mean ± SD. Data were evaluated by unpaired Student’s *t*-test between two independent groups. Survival curves were assessed by log-rank (Mantel-Cox) tests. *P* < 0.05 was considered significant. Adobe Illustrator 2020, Adobe Photoshop 2020, and Image J software were used for the stitching of pictures.

## Results

### CRC-CDEs promote cell migration, tube formation, and filopodia formation of HUVECs

**Fig. 1:**
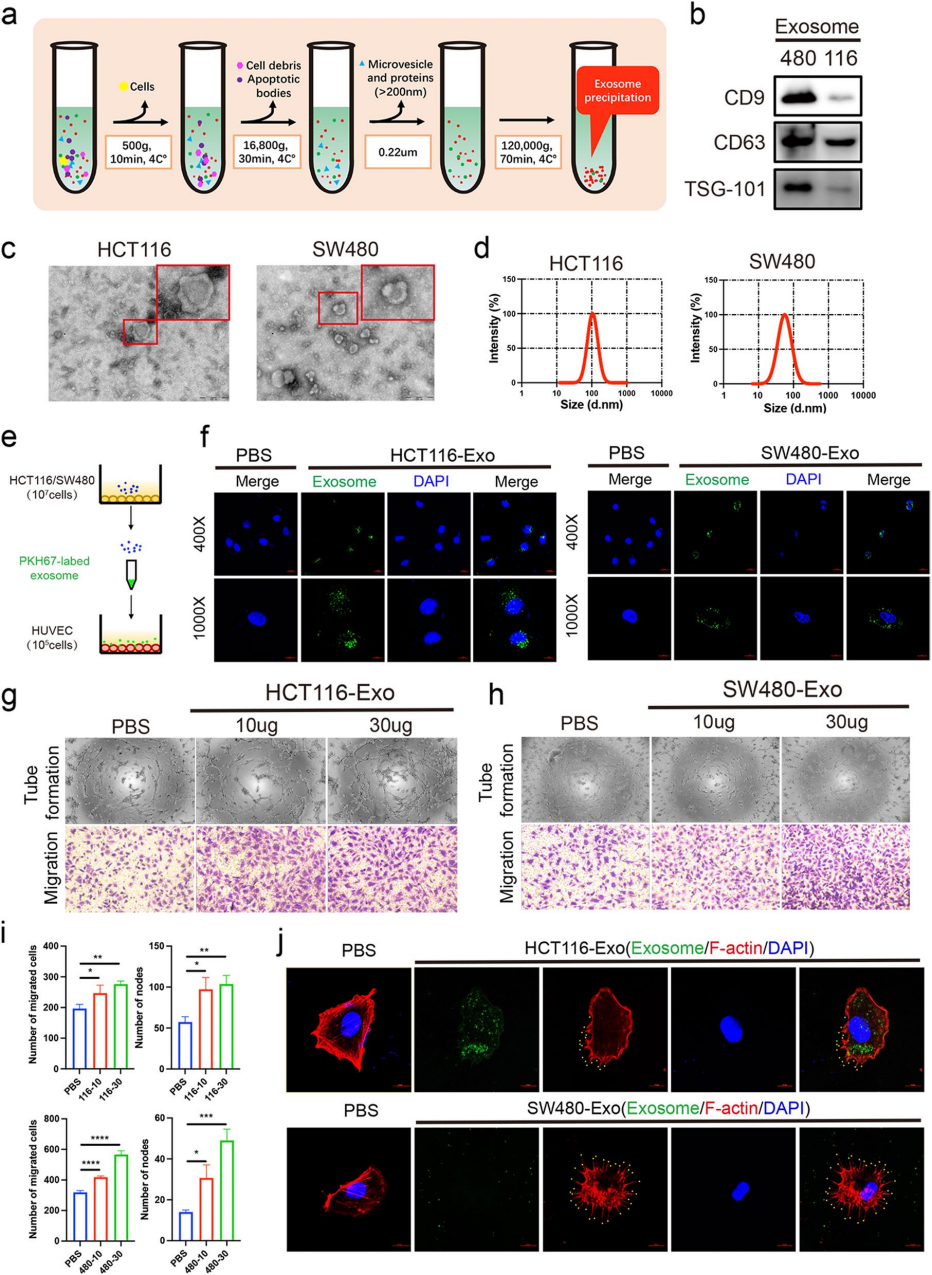
CRC-CDEs promote cell migration, tube formation, and filopodia formation of HUVECs. **a** Diagram of ultracentrifugation method. **b** Western blot of non-lysed HCT116-Exo and SW480 protein (CD9, CD63, and TSG-101). **c** TEM images of obtained exosome from the cell culture supernatant of HCT116 and SW480 (HCT116-Exo and SW480-Exo). Scale bar = 200 nm. **d** NTA analysis of size distribution and number in HCT116-Exo and SW480-Exo. **e** Schematic description of exosome labeled by PKH67 membrane dye. HCT116-Exo and SW480-Exo from 1 × 10^7^ HCT116 were isolated and cultured with 1 × 10.^5^ HUVECs. **f** Confocal microscopy image of PKH67-labeled exosome in HUVECs after 3 h. Scale bars = 10 μm and 20 μm. HUVECs incubated with PBS were used as a negative control. Migration (**g**) and Tube formation (**h**) of HUVECs cultured with exosome (10ug or 30ug) from HCT116 (116) and SW480 (480) after 48 h. HUVECs incubated with PBS were used as a negative control. **i** The number of migrated cells and nodes was analyzed by Image J. **h** Confocal microscopy image of phalloidin-labeled F-actin and PKH67-labeled exosome in HUVECs after 24 h. Scale bars = 10 μm. Mean ± SEM. Student's t-test, *P < 0.05, 0.001 < ** *P* < 0.01, *** *P* < 0.001

To study the role of CRC-CDEs in angiogenesis, we first executed exosome isolation and identification. Exosomes derived from the supernatant of HCT116 cells and SW480 cells were isolated by differential centrifugation (Fig. 1a). The biomarkers of exosomes (CD9, CD63, TSG-101) were detected by Western blotting in HCT116-Exo and SW480-Exo without lysis (Fig. 1b). Furthermore, TEM analysis revealed that these vesicles of HCT116 and SW480 were globular and had a typical cup shape. Moreover, HCT116-Exo was bigger than SW480-Exo (Fig. 1c). The NTA results showed the average size of HCT116-Exos was also higher than SW480-Exo (Fig. 1d). Then, the PKH67-labeled exosome from 1 × 10^7^ HCT116 cells was added to HUVECs (Fig. 1e). Confocal imaging showed HCT116-Exo and SW480-Exo accumulated in the cytoplasm which suggested the exosome was taken up by HUVECs at 3 h (Fig. 1f). To ensure the role of HCT116-Exo and SW480-Exo, HUVECs were incubated with 10 μg or 30 μg exosome. After 48 h, transwell assays revealed that the different concentrations of HCT116-Exo and SW480-Exo treatment significantly enhanced HUVECs migration and tube formation compared with PBS treatment (Fig. 1g-i). A similar trend was displayed in the SW480-Exo treatment group (Fig. 1h-i). To determine whether the effect of exosomes on migration was due to altered HUVECs morphology, we used phalloidin to label F-actin in HUVECs. Interestingly, confocal imaging revealed filopodial extensions (yellow dot mark) in HUVECs which absorbed HCT116-Exo and SW480-Exo (Fig. 1j). These results display that exosome derived from CRC cells enhances HUVECs' capability of migration and tube formation, suggesting that recipient cells can be remodeled by CRC-CDEs.

### CRC-CDEs promote tip cell formation of HUVECs

**Fig. 2:**
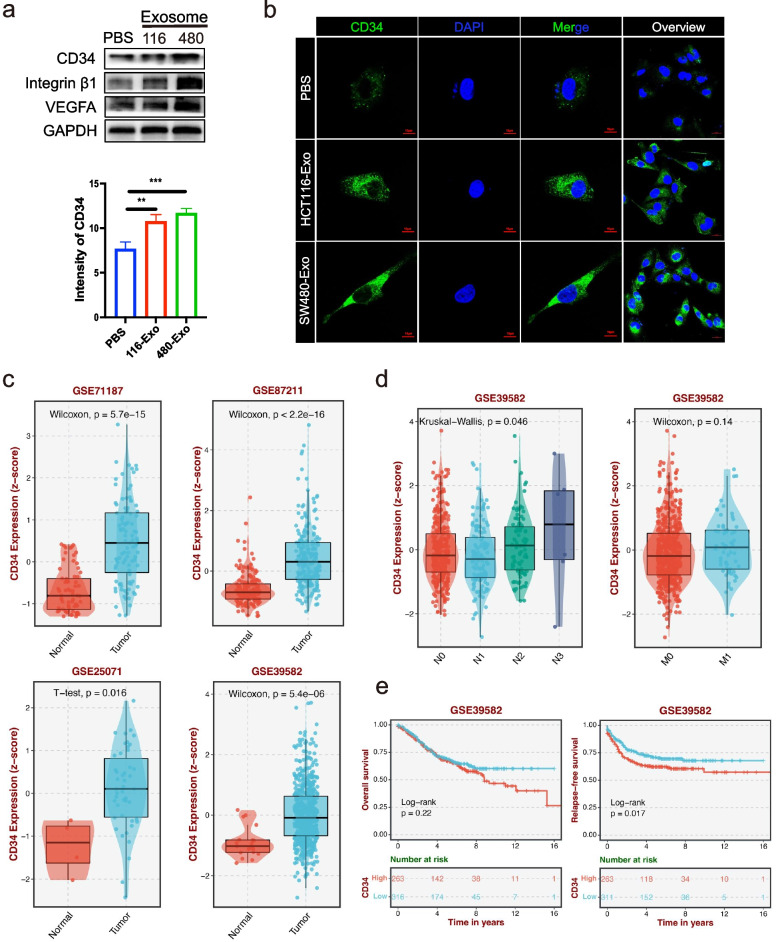
CRC-CDEs promote tip cell formation of HUVECs. **a** Western blot detection of CD34, integrin β1, and VEGFA in lysis of HUVECs cultured with HCT116-Exo and SW480-Exo after 48 h, GAPDH was the internal control of whole-cell lysates. The PBS group was a negative control. **b** Confocal microscopy image of CD34-immunofluorescence in HUVECs cultured with HCT116-Exo and SW480-Exo after 48 h. Scale bars = 10 μm and 20 μm. The intensity of CD34 (right) was measured by Zeiss Zen software. Mean ± SEM. Student's t-test, 0.001 < ** *P* < 0.01, *** *P* < 0.001. HUVECs incubated with PBS were used as a negative control. **c** CD34 expression in CRC was analyzed from the data of GSE71187, 87211, 25071, and 39582. Statistical significance was calculated by Student's t-test and Wilcoxon. **d** CD34 expression in the different stages of lymph node metastasis (N0, N1, N2, N3) and distant metastasis (M0, M1) were analyzed from the data of GSE39582. Statistical significance was calculated by Kruskal–Wallis and Wilcoxon. **e** The OS and RFS of CD34 in CRC were analyzed from the data of GSE39582. Log-rank test was used to estimate the significance. All data of GSE71187, 87211, 25071, and 39582 were reanalyzed using the BEST (https://rookieutopia.com/)

Based on the results of HUVECs morphology changing by CRC-CDEs, we speculate that CRC-CDEs may induce sprout formation and tip cell differentiation. Integrin β1 and VEGFA are reported to be involved in vessel growth and maturation and are required for efficient endothelial sprout formation [12–14]. We found that high HCT116-Exo and SW480-Exo treatment led to high integrin β1 and VEGFA expression compared with PBS treatment (Fig. 2a). CD34 is a well-recognized maker of tip cells that showed filopodial extensions to promote angiogenic sprouts [15]. Therefore, we detected the CD34 expression by immunofluorescence and western blots after CRC-CDEs treatment. The results of western blot displayed high CD34 expression after HCT116-Exo and SW480-Exo treatment compared with PBS treatment (Fig. 2a). The immunofluorescence image showed that the intensity of CD34 in HCT116-Exo and SW480-Exo groups was higher than in PBS groups (Fig. 2b). To ensure CD34 expression in the CRC sample, the GEO datasets were used to analyze. The analysis results (GSE71187, GSE87211, GSE25071, GSE39582) showed that CD34 was significantly high-expressed in CRC tissue compared with normal tissue (Fig. 2c). Moreover, we found that CD34 expression in GSE39582 was gradually elevated in the N1, N2, and N3 groups. High CD34 expression was also found in the M1 group compared to the M0 group and showed a worse overall survival (OS) and relapse-free survival (RFS) (Fig. 2d and e). In addition, we found that CD34 expression was slightly upregulated in the bevacizumab-resistant group compared non-resistant group (Fig. S1a). Moreover, high ITGB1(Integrin β1) expression was found in CRC tissues compared to normal tissues and predicted a poor prognosis of CRC patients (Fig. S1b). These data suggest CRC-CDEs promote tip cell formation via upregulating VEGFA, integrin β1, and CD34 expression, which predicts CRC metastasis and a poor prognosis for CRC patients.

### CircRNA are enriched in exosomes secreted by CRC cells and serum of CRC patients

**Fig. 3:**
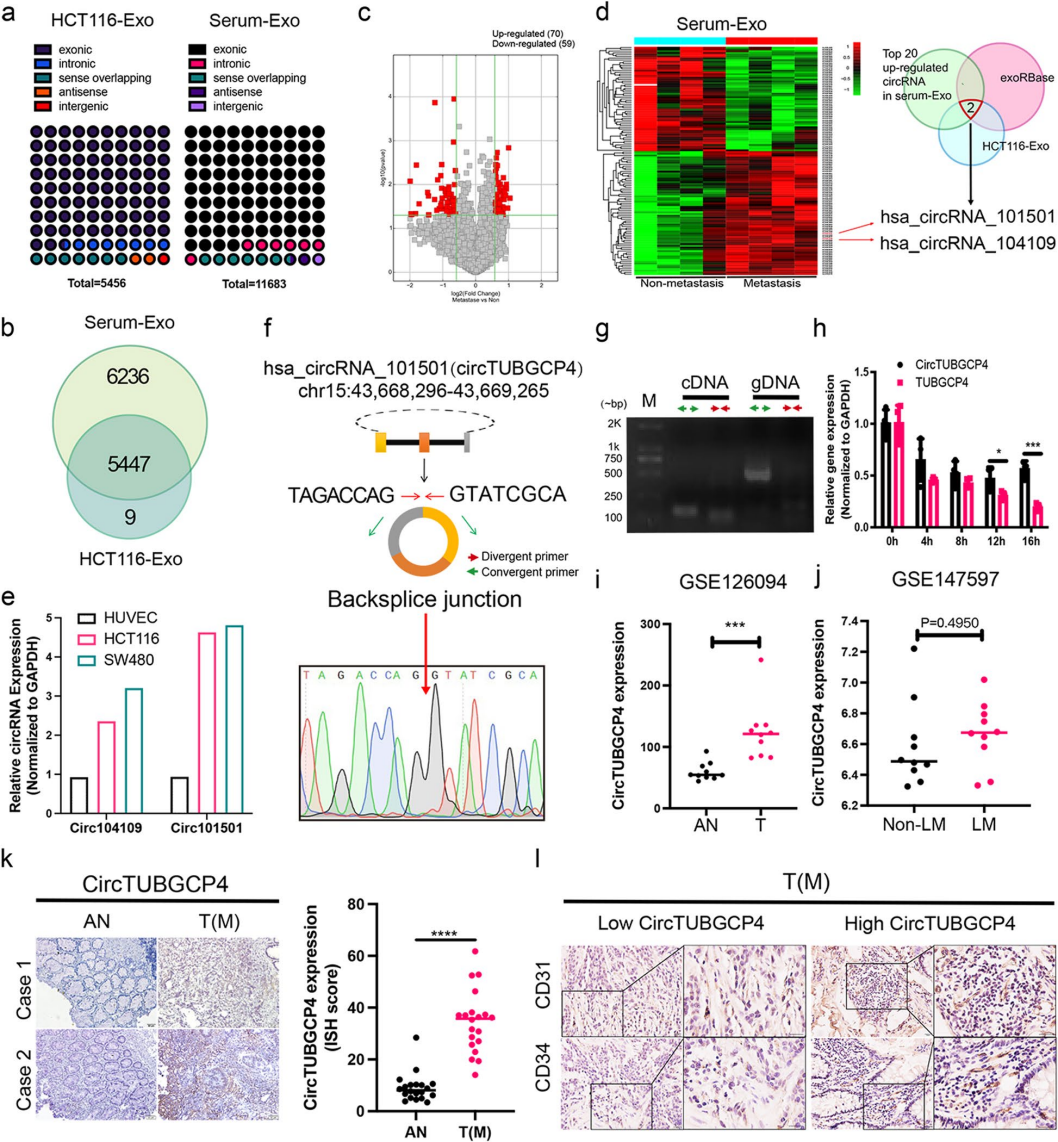
CircTUBGCP4 in CRC-CDEs was associated with angiogenesis and metastasis in CRC. **a** The percent of different circRNA types in HCT11-Exo and serum-Exo, each point represents 1%. **b** Venn diagram of circRNA identified in HCT11-Exo and serum-Exo. **c** The volcano map of differently expressed circRNA in serum-Exo from CRC patients with metastasis or non-metastasis. *p* < 0.05, fold change > 1.5 or fold change < -1.5. **d** The screen flow of circRNA by three databases that circRNA in HCT116-Exo, Top 20 up-regulated circRNA in serum-Exo, and exoRBase. **e** The expression of circ104109 and circ101501 in HUVECs, HCT116, and SW480. GAPDH was the internal control. **f** The detail of circTUBGCP4 and Sanger sequence of amplification products using a divergent primer. **g** Agarose gel analysis of PCR production using divergent primer and convergent primer of circTUBGCP4. **h** qPCR analysis of circTUBGCP4 and TUBGCP4 expression after HCT116 cells culture with actinomycin D at 0, 4, 8, 12, and 16 h. GAPDH was the internal control. **i** GSE126094 reanalysis of circTUBGCP4 expression in CRC T compared with AN. **j** CircTUBGCP4 expression in CRC LM compared with CRC non-LM using GSE147597 data. **k** ISH analysis of circTUBGCP4 in the clinical cohort from 20 pairs CRC T(M) and AN. Scale bars = 50 μm. **l** Correlation analysis between circTUBGCP4 expression and CD31, CD34 expression in tumor tissue. Student's t-test, *P < 0.05, 0.001 < ** *P* < 0.01, *** *P* < 0.001

It has been reported that circRNA is enriched and stable in exosomes and can be a promising marker for cancer diagnosis [8]. To study which components in CRC-CDEs promote angiogenesis to accelerate cancer metastasis, we isolated HCT116-Exo and serum exosomes from CRC patients with or without metastasis. Next, the differential circRNA expression profile of these exosomes was investigated by circular RNA microarray. A total of 5456 and 11,683 circRNAs were identified in serum-Exo and HCT116-Exo (Fig. 3a). About 84.2% of circRNAs in serum-Exo were derived from exonic sequence and about 15.8% were derived from intronic, sense overlapping, antisense, and intergenic sequence (Fig. 3a). Moreover, about 82.7% of circRNAs in HCT116-Exo were exonic circRNA and about 17.3% were other types of circRNA, including intronic, sense overlapping, antisense, and intergenic sequence (Fig. 3a). The result showed that circRNA in serum-Exo and HCT116-Exo are enriched and of various types. A total of 5447 circRNAs were simultaneously identified in serum-Exo and HCT116-Exo (Fig. 3b). It means that about 46.6% of circRNA in serum-Exo are from cancer cells (Fig. 3b). The result showed most circRNA in serum-Exo are present in CRC-CDEs. These results suggest an enormous potential of circRNA as tumor markers and the complex regulatory mechanism of tumor microenvironment changed by circRNA in CRC-CDEs.

### Upregulated circTUBGCP4 was associated with angiogenesis and metastasis in CRC

To study the role of circRNA from CRC-CDEs in promoting angiogenesis to drive tumor metastasis, we compared the expression levels of circRNA in serum exosomes from CRC patients with or without metastasis. There were 70 up-regulated circRNAs and 59 down-regulated circRNAs in serum exosome with metastasis compared non-metastasis group (Fig. 3c, *p*< 0.05, fold change > 1.5 or fold change < -1.5). The exoRbase which is a database of circRNA, lncRNA and mRNA in human blood exosomes was used for the circRNA screen [16]. Then, exonic hsa_circRNA_101501 and has_circRNA_104109 were chosen by three datasets including HCT116-Exo, exoRBase, and the top 20 most highly expressed circRNAs of serum-Exo (Fig. 3d). Compared to HUVECs, the fold change of circ101501 expression in HCT116 and SW480 was higher than circ104109 (Fig. 3e). Therefore, we chose the circ101501 and renamed it circTUBGCP4, which its parent gene is TUBGCP4.

CircTUBGCP4 was composed of three exons from chr15:43,668,296–43,669,265. Firstly, we identified the ring structure of circTUBGCP4 by sanger sequence, verification by divergent primer (DP) and convergent primer (CP), and Actinomycin D assay. The result of the sanger sequence showed that the PCR products contained the sequence of backsplice junctions using a divergent primer (Fig. 3f). The DP could amplify products when cDNA was used as a PCR template, but not when gDNA was used as a template (Fig. 3g). Actinomycin D assay showed that circTUBGCP4 was more stable than TUBGCP4 (Fig. 3h). These results prove that circTUBGCP4 is a ring structure. To explore the circTUBGCP4 expression and its association with angiogenesis and metastasis in CRC, we used the GEO dataset (GSE126094 and GSE147597) and our clinical cohort (*n* = 20 pairs) for analysis. The GSE126094 data showed circTUBGCP4 was significantly upregulated in primary tumor compared with its adjacent normal tissue (AN) (Fig. 3i). High circTUBGCP4 expression was found in liver metastasis (LM) compared with non-liver metastasis (Non-LM) in GSE147597 (Fig. 3j). Furthermore, the ISH results of the clinical cohort revealed that circTUBGCP4 in primary tumor with lymph node metastasis was higher than adjacent normal tissue (Fig. 3k). In addition, we found high CD31 and CD34 expression in the high circTUBGCP4 group compared with the low circTUBGCP4 group (Fig. 3l). These results suggest that high circTUBGCP4 expression may be related to tumor angiogenesis and metastasis.

### Silencing exosomal circTUBGCP4 inhibited tip cell formation, angiogenesis, and tumor metastasis

**Fig. 4:**
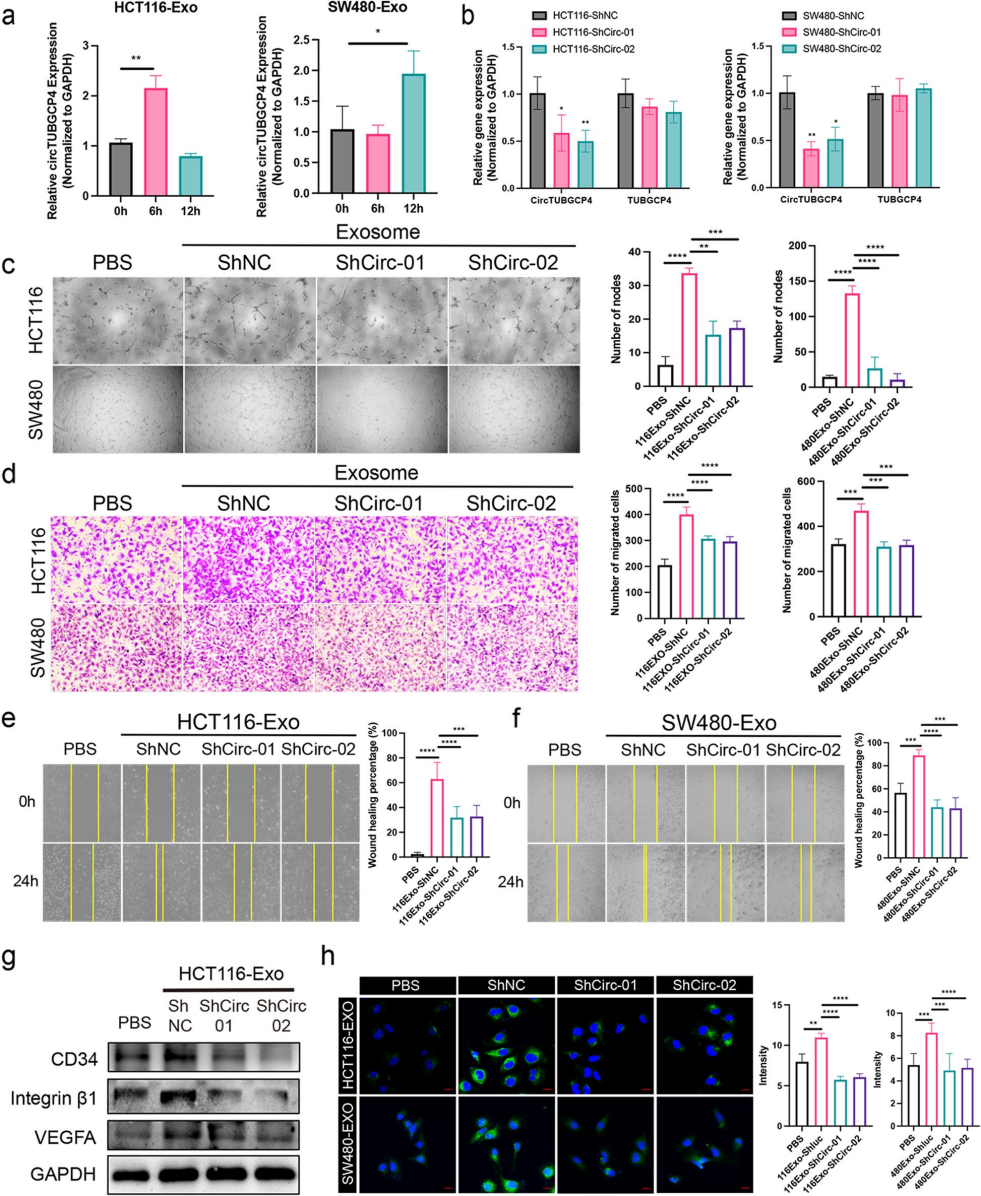
Exosomal circTUBGCP4 promotes HUVECs tip cell formation, migration, and tube formation. **a** CircTUBGCP4 expression was detected in HUVECs treated with HCT116-Exo and SW480-Exo at 0 h, 6 h, and 12 h. **b** The stable silencing effect and specificity of circTUBGCP4 in HCT116 and SW480. Effect of exosome of shcircTUBGCP4-01 (shCirc-01) and shcircTUBGCP4-02 (shCirc-01) derived from HCT116 and SW480 stable cell lines on tube formation ability and migration ability of HUVECs using tube formation assay (**c**), migration transwell assay (**d**), and wound healing assay (**e** and **f**). Then wound healing percentage (%) and the number of migrated cells and nodes were analyzed by Image J and Prism 9. **g** The CD34, integrin β1, and VEGFA expression of HUVECs treated with HCT116-exosome derived from shcircTUBGCP4-01 and shcircTUBGCP4-02 stable cell lines were detected by Western blot. GAPDH was the internal control of whole-cell lysates. **h** The CD34 expression of HUVECs treated with HCT116-exosome and SW480-exosome derived from shCirc stable cell lines were detected by immunofluorescence using a confocal microscopy image. HUVECs incubated with PBS and shNC-Exo was used as a negative control. Mean ± SEM. Student's t-test, *P < 0.05, 0.001 < ** *P* < 0.01, *** *P* < 0.001

**Fig. 5:**
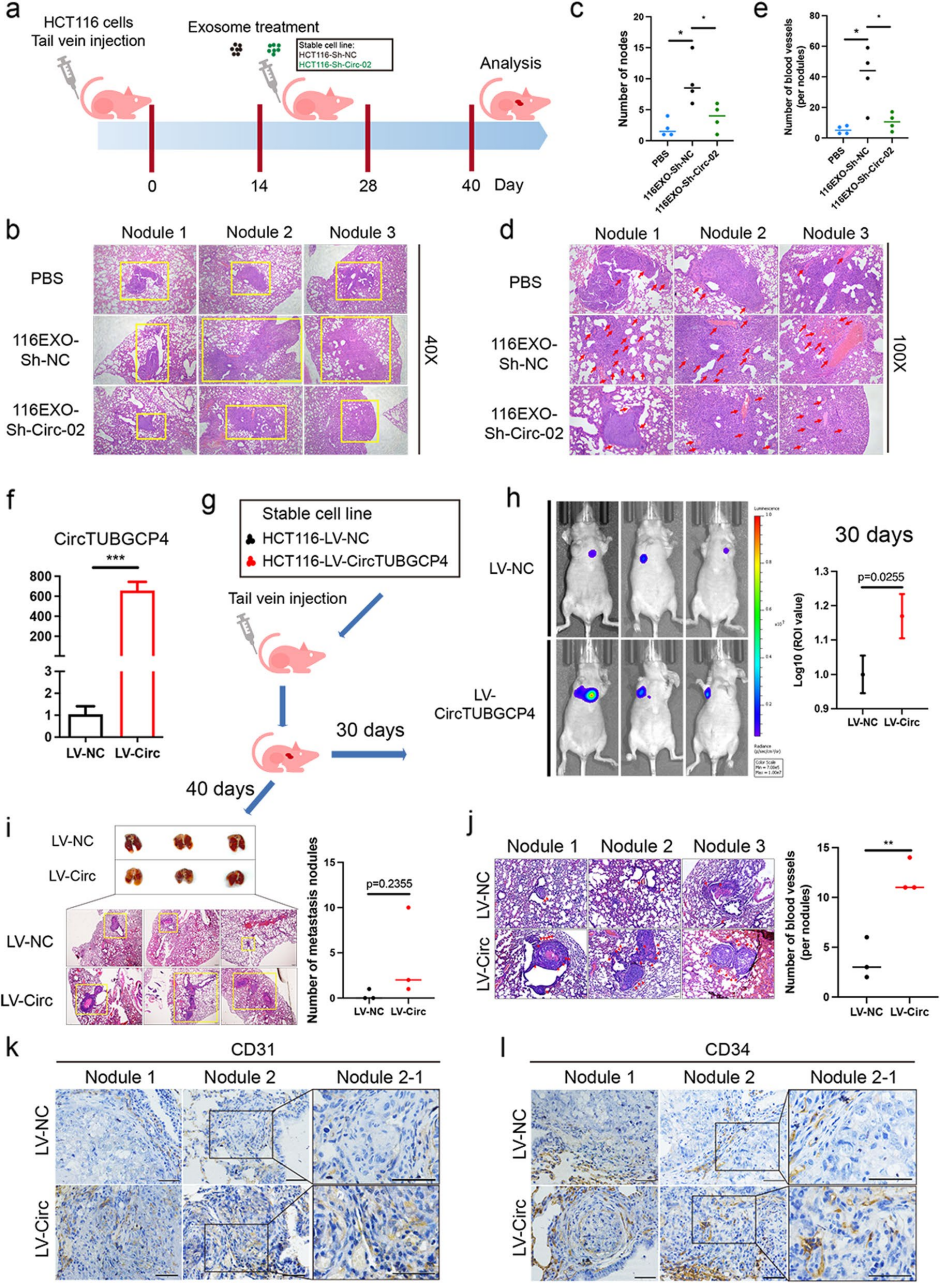
Overexpressed circTUBGCP4 promotes angiogenesis and tumor metastasis. **a** A flowchart of lung metastasis model building using exosome derived from HCT116- ShCirc stable cell lines in vivo. Exosomes (40 μg) are injected every 4 days for 12 days. PBS was used as a negative control. **b** Lung metastasis nodules in HCT116EXO-Sh-NC (116EXO-Sh-NC) groups, HCT116EXO-Sh-CircTUBGCP4-02 (116EXO-Sh-Circ-02) groups compared to PBS groups using HE staining. Scale bars = 200 μm. **c** The number of metastasis nodules was counted. **d** Blood vessels in lung metastasis nodule (red arrows) using HE staining. Scale bars = 100 μm. **e** The number of blood vessels (per nodule) was counted. **f** The efficiency of overexpressed circTUBGCP4 in HCT116 stable cell line. **g** Flow chart of lung metastasis model building using LV-NC HCT116 cells and LV-CircTUBGCP4 HCT116 cells in vivo. **h** In vivo fluorescence imaging of the metastasis model at 30 days. **i** Lung metastasis nodules in LV-CircTUBGCP4 compared to LV-NC using HE staining. Scale bars = 200 μm. The number of blood vessels (per nodule) was counted. **j** Blood vessels in lung metastasis nodule (red arrows) using HE staining. Scale bars = 100 μm. The number of blood vessels (per nodule) was counted. **k** and (**i**) Immunohistochemistry analysis of CD31 expression and CD34 expression in lung metastasis nodule, Scale bars = 20 μm. Mean ± SEM. Student's t-test, *P < 0.05, 0.001 < ** *P* < 0.01, *** *P* < 0.001

To explore the biological function of exosomal circTUBGCP4 in HUVECs, we first should ensure the exosomal circTUBGCP4 could be absorbed by HUVECs. We isolated HCT116-Exo and SW480-Exo to incubate HUVECs for 6 h, and 12 h. We found that circTUBGCP4 was upregulated in HCT116-EXO-6 h group and SW480-EXO-6 h group compared to the group without exosome treatment (Fig. 4a). These results verify that tumor cell-derived circTUBGCP4 maybe enter HUVECs via assembling into exosomes. Next, we constructed stable cell lines of circTUBGCP4 knockdown in HCT116 and SW480. The results showed that circTUBGCP4 was significantly down-regulated in shcircTUBGCP4-01 and shcircTUBGCP4-02 group compared to shNC group while its parental gene was unchanged (Fig. 4b). Then, we extract exosomes from stable cell lines to study the biological effect on HUVECs. Tube formation assays revealed that knockdown exo-circTUBGCP4 significantly decreased the number of nodes in HUVECs (Fig. 4c). Transwell assays showed that downregulated exo-circTUBGCP4 reduced the migration capacity (Fig. 4d). Cell scratches assay displayed a slow healing speed in shcircTUBGCP4-01 and shTUBGCP4-02 group compared to shNC group (Fig. 4e-f). The expression of CD34, integrin β1, and VEGFA downregulated in HUVECs treated with shcircTUBGCP4-Exo using western blot (Figs. 4f and S2a). Moreover, immunofluorescence results showed a low CD34 expression in the group of Exo-ShCirc-01 and ShCirc-01 derived from HCT116 and SW480 (Figs. 4g and S2b). To evaluate the potential contribution of exosomal circTUBGCP4 to angiogenesis and tumor metastasis in vivo, we first injected HCT116 cells and two weeks later injected exosomes from Sh-NC and Sh-Circ-02 groups through the tail vein (Fig. 5a). The result showed that HCT116Exo-Sh-NC led to an increase in lung nodes compared to PBS and HCT116Exo-Sh-Circ-02 (Fig. 5b-c). Moreover, the number of blood vessels in the lung node was increased in HCT116Exo-Sh-NC and decreased in HCT116Exo-Sh-Circ-02 (Fig. 5d-e). These results prove that exosomal circTUBGCP4 can induce tip cell formation, angiogenesis, and tumor metastasis.

### Overexpressed circTUBGCP4 promotes tip cell formation, angiogenesis, and tumor metastasis

To evaluate the potential contribution of circTUBGCP4 effect in vitro and in vivo, we first construct overexpression plasmid and lentivirus of circTUBGCP4 (Fig. S3a). The results of the transwell and tube formation assay displayed that HUVECs transfected circTUBGCP4 plasmid increased the number of migration and nodes (Fig. S3b). Moreover, we found that overexpressed circTUBGCP4 promoted the expression of Integrin β1 and VEGFA (Fig. S3c). Next, we verified the overexpression efficiency of the stable cell line and constructed a tail vein model (Fig. 5f and g). In vivo fluorescence imaging results showed a stronger fluorescence in mice lungs of LV-CircTUBGCP4 group compared with LV-NC after 30 days (Fig. 5h). Moreover, the number of lung metastasis nodules in LV-CircTUBGCP4 group was more than LV-NC group (Fig. 5i). In addition, HE results and IHC results revealed LV-CircTUBGCP4 group had more blood vessels and microvascular than LV-NC group (Fig. 5j-l). Interestingly, the LV-CircTUBGCP4 group showed higher CD31 + and CD34 + in lung metastasis nodules compared with the LV-NC group (Fig. 5k-l). These results indicate that overexpressed circRNAs can enhance angiogenesis to promote tumor metastasis.

### CircTUBGCP4 activates Akt signaling pathway by targeting miR-146b-3p/PDK2 axis in HUVECs

**Fig. 6:**
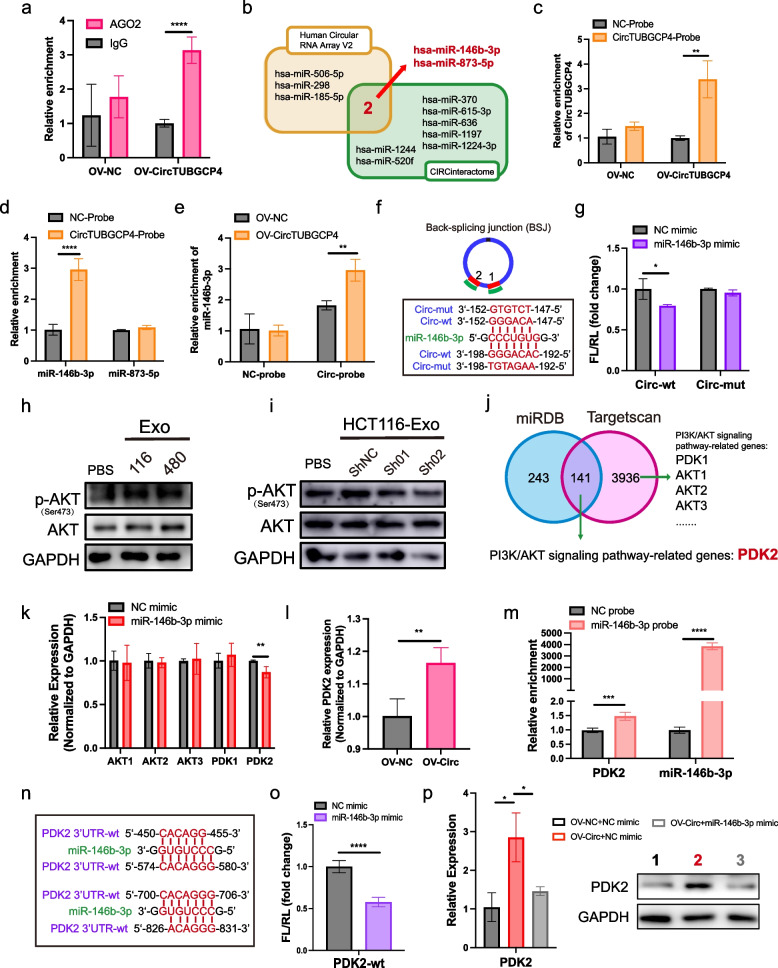
CircTUBGCP4 sponge miR-146b-3p to upregulate PDK2 for activating Akt signaling pathway in HUVECs. **a** AGO2-RIP analysis of the binding between circTBUGCP4 and AGO2 in HUVECs transfected overexpressed circTUBGCP4 plasmid and OV-NC plasmid. **b** MiRNA screening from circular RNA array analysis and the CIRCinteractiome website. **c** The efficiency of circTUBGCP4 probe by circRNA pull-down assay in HUVECs transfected the plasmid of OV-CircTUBGCP4 and OV-NC. NC probe was the negative control. **d** qPCR analysis of circRNA-pulled down miR-146b-3p and miR-873-5p in HUVECs transfected OV-CircTUBGCP4 plasmid. **e** Enrichment of miR-146b-3p pulled down by circTUBGCP4 probe compared with NC probe in HUVECs transfected OV-CircTUBGCP4 plasmid and OV-NC plasmid. f MiR-146b-3p binding sites in circTUBGCP4. **g** Luciferase activity of 293T transfected with circTUBGCP4-wt/mu luciferase constructs with miR-146b-3p mimic or negative control. **h** p-AKT (Ser 473) and AKT expression in HCT116-Exo and SW480 Exo-treated HUVECs after 48 h using western blot. **i** Expression of p-AKT (Ser 473) and AKT in HUVECs treated with exosomes derived from shcircTUBGCP4-01, shcircTUBGCP4-02, and shNC. **j** Venn diagram of potential targets of miR-146b-3p using miRDB and Targetscan. **k** qPCR confirmation of possible targets in HUVECs transfected with miR-146b-3p mimic and NC mimic. **l** Expression of PDK2 mRNA in OV-circTUBGCP4 group and OV-NC group of HUVECs detected by qPCR. **m** Binding verification of PDK2 mRNA and miR-146b-3p by pull-down assay using biotin-labeled miR-146b-3p in HUVEC, NC probe for miR-146b-3p was as a negative control. **n** The binding site of miR-146b-3p in PDK3 mRNA-3’UTR. **o** Luciferase activity of 293 T transfected with PDK2-3’UTR luciferase constructs with miR-146b-3p mimic or negative control. **p** Rescue experiment using OV-circTUBGCP4 plasmid and miR-146b-3p mimic for detecting PDK2 expression in mRNA level and protein by qPCR and western blot. GAPDH was the internal control of whole-cell lysates. Mean ± SEM. Student's t-test, *P < 0.05, 0.001 < ** *P* < 0.01, *** *P* < 0.001

To explore the underlying regulation of exosomal circTUBGCP4, we considered whether circTUBGCP4 acts as sponges to adsorb miRNAs. Firstly, we used the Cancer-specific circRNAs database (http://gb.whu.edu.cn/CSCD/) and find eight AGO2-binding sites in circTUBGCP4 (Table S4). Moreover, AGO2-RIP verified that circTBUGCP4 could bind AGO2 protein which suggested the potential of circTUBGCP4 binding miRNA (Fig. 6a). Then, we chose miR-146b-3p and miR-873-5p from the intersection of our circular RNA array analysis and the CIRCinteractiome (https://circinteractome.nia.nih.gov/) (Fig. 6b). We designed biotin probes for circTUBGCP4 targeting the back splicing junction and verified the good pull-down efficiency in HUVECs transfected overexpression circTUBGCP4 (Fig. 6c). The circRNA pull-down results showed that the circTUBGCP4 probe could significantly bind miR-146b-3p, not miR-873-5p in the HUVEC lysis buffer (Fig. 6d). Moreover, the circTUBGCP4 probe could pull more miR-146b-3p when HUVECs transfected overexpress circTUBGCP4 (Fig. 6e). Next, we found that circTUBGCP4 had two sites to sponge miR-146b-3p (Fig. 6f). Based on two binding sites, we constructed a full mutant plasmid of circTUBGCP4. The results showed circTUBGCP4 could bind miR-146b-3p based on the two sites in 293 T (Fig. 6g).

It has been reported that cancer cell co-culture with HUVECs could increase endothelial cell tube formation and survival by activating PI3K/Akt signaling pathway [17]. Therefore, we wondered whether exosomal circTUBGCP4 promoted endothelial cell tube formation by activating the Akt pathway. The Western blot results revealed upregulated p-AKT in HUVECs treated with HCT116-Exo and SW480-Exo, and a decrease of p-AKT in HUVECs treated with shcircTUBGCP4-01-Exo, and shcircTUBGCP4-02-Exo (Fig. 6h and i). Therefore, we screen the target of miR-146b-3p related to PI3K/Akt signaling pathway. Coincidentally, among the 141 target genes screened from Targetscan and miRDB databases, PI3K/Akt signaling pathway-related gene is PDK2 (Fig. 6j). In addition, we also screened other primary genes directly related PI3K/Akt signaling pathway (Fig. 6j). Then, we found that PDK2 was the only downregulated target in HUVECs overexpressed miR-146b-3p compared with NC (Fig. 6k). Moreover, PDK2 could be upregulated by circTUBGCP4 and downregulated by Exo-Sh-Circ-01 and Exo-Sh-Circ-02 of HCT116 and SW480 (Figs. 6l, S2c). To prove miR-146b-3p targeting PDK2-3’UTR, we designed the miR-146b-3p biotin probe to pulldown PDK2. The results showed that the miR-146b-3p probe could markedly enrich PDK2 (Fig. 6m). Then, we constructed a PDK2-3’UTR plasmid that carried a dual luciferase reporter gene, and we forecasted its binding site in PDK2-3’UTR in 293 T cells (Fig. 6n). The results displayed that PDK2-3’UTR could be bound by miR-146b-3p in 293 T cells (Fig. 6o). To confirm circTUBGCP4 activating Akt signaling pathway by miR-146b-3p, we performed transfection in HUVECs when circTUBGCP4 was overexpressed while overexpression of miR-146b-3p (Fig. S4a). The results showed that PDK2 in mRNA level and protein level were increased in overexpressed-circTUBGCP4 HUVECs. Then the increase was inhibited by miR-146b-3p mimic in HUVECs (Fig. 6p). Collectively, these results suggest that circTUBGCP4 can sponge miR-146b-3p to promote PDK2 for activating Akt signaling.

### MiR-146b-3p inhibits HUVECs migration, tube formation, and tip cell formation

**Fig. 7:**
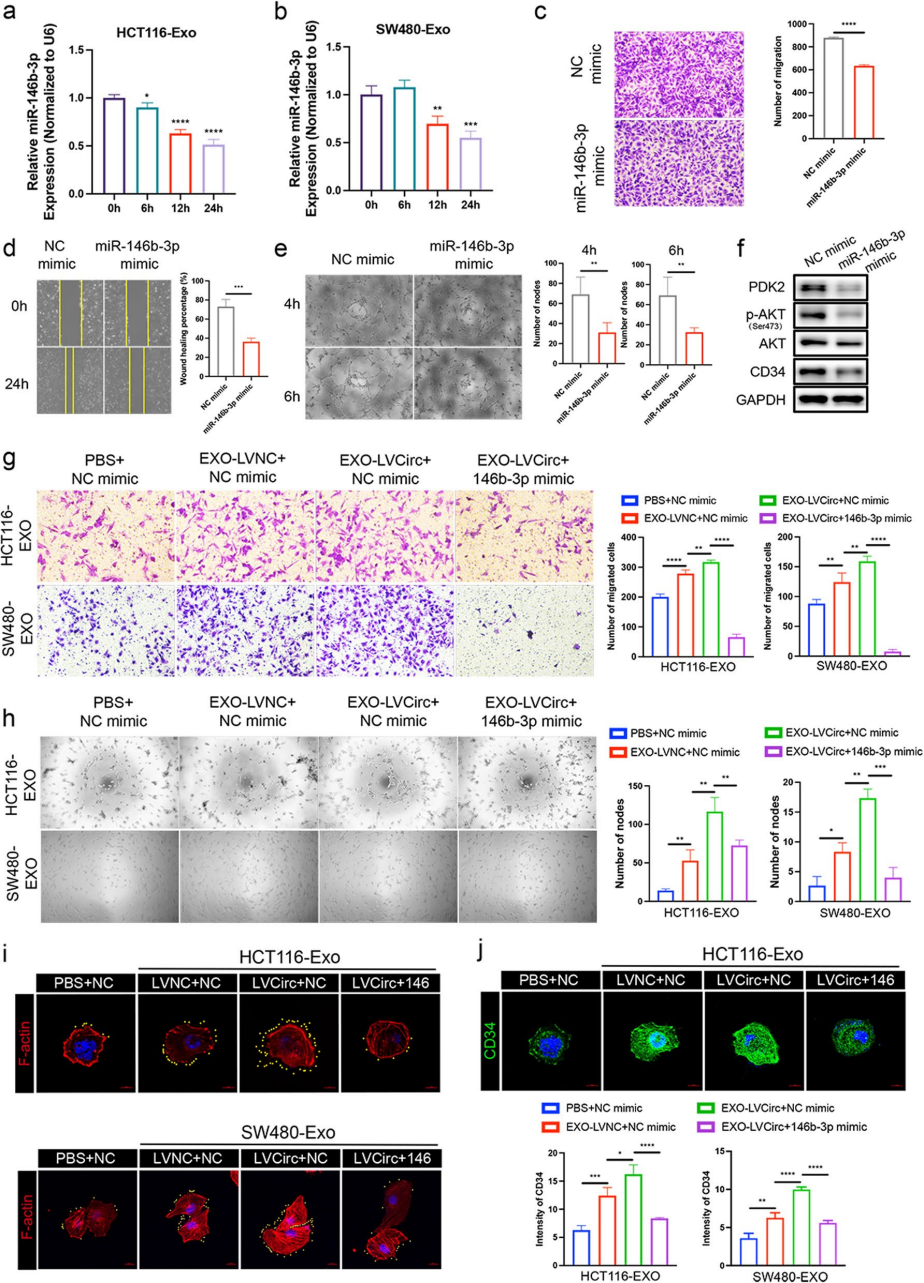
The role of miR-146b-3p in HUVECs, and exosomal circTUBGCP4 contribution to HUVECs dysfunction regulated by miR-146b-3p. (**a** and **b**) miR-146b-3p expression in HUVECs treated with HCT116-Exo and SW480-Exo at 0 h, 6 h, 12 h, and 24 h. HUVECs were transfected with miR-146b-3p mimic and NC mimic for 48 h. Then, the migration and tube formation ability of HUVECs were evaluated by transwell migration assay (**c**), wound healing assay (**d**), and tube formation assay (**e**), then wound healing percentage (%) and the number of migrated cells and nodes were analyzed by Image J and Prism 9. (**f**) Western blot analysis of PDK2, p-AKT (Ser473), AKT and CD34 in HUVECs transfected with miR-146b-3p mimic and NC mimic. GAPDH was the internal control of whole-cell lysates. HUVECs were treated with exosomes derived from LVcircTUBGCP4-Exo (HCT116 and SW480) and its negative control, follow by transfected with miR-146b-3p mimic and NC mimic. After 48 h, the migration and tube formation were measured by transwell migration assay (**g**) and tube formation assay (**h**). The number of migrated cells and nodes was analyzed by Image J and Prism 9. (**i**) Confocal microscopy image of F-actin (red) in HUVECs treated with exosome derived from LVcircTUBGCP4-Exo (HCT116 and SW480) and its negative control, follow by transfected with miR-146b-3p mimic and NC mimic, Scale bars = 10 μm. The yellow dot is used to mark the filopodia represented by F-actin. (**j**) CD34 immunofluorescence in the above groups, the intensity of CD34 was analyzed by Zeiss Zen software (left), Scale bars = 10 μm. Mean ± SEM. Student's t-test, *P < 0.05, 0.001 < ** *P* < 0.01, *** *P* < 0.001

Next, based on the above findings, we detected the expression of miR-146b-3p after HCT116-Exo and SW480-Exo treatment to HUVECs at 6 h, 12 h, and 24 h. As shown in Fig. 7a-b, miR-146b-3p was gradually decreased by HCT116-Exo and SW480-Exo. To confirm the potential function of miR-146b-3p in HUVECs, the miR-146b-3p mimic was transfected in HUVECs (Fig. S4b). Then, the transwell migration assay showed that overexpressed miR-146b-3p significantly inhibited HUVECs migration (Fig. 7c). Additionally, scratch wounding assays also displayed a slow wound closure of HUVECs in the miR-146b-3p mimic group compared with the NC mimic group (Fig. 7d). Furthermore, tube formation assay revealed that overexpressed miR-146b-3p markedly inhibitor nodes forming of HUVECs after 4 h and 6 h (Fig. 7e). Then, based on the results of western blot, we found that miR-146b-3p could significantly inhibit CD34 expression, but also the expression of PDK2 and the activation of AKT (Fig. 7f).

We next analyzed the expression of miR-146b-3p in TCGA using UALCAN (http://ualcan.path.uab.edu/). The dataset showed that miR-146b-3p was downregulated in CRC primary tumors compared with normal tissue (Fig. S5a). Moreover, miR-146b-3p expression was significantly decreased at stage1, 3, and 4 (Fig. S5b). Furthermore, gradual low miR-146b-3p expression was observed in N0, N1, and N2 (Fig. S5c). These data suggest that miR-146b-3p can be an important regulator in inhibiting HUVECs migration, tube formation, and tip cell formation via Akt signaling pathway, and is dramatically associated with tumor metastasis.

### Exosomal circTUBGCP4 enhances HUVECs migration, tube formation, and tip cell formation by inhibiting miR-146b-3p

To explore the underlying role of miR-146b-3p on HUVECs function regulated by circTUBGCP4, we used exosome from HCT116-LV-CircTUBGCP4 to incubate HUVECs which was subsequently transfected with miR-146b-3p mimic. Then, transwell migration assay revealed an enhanced migration ability of HUVECs treated with Exo-LVcircTUBGCP4 compared with Exo-LVNC of HCT116 and SW480, then the increasing trend was inhibited by miR-146b mimic (Fig. 7g). A similar result of tube formation assay showed that HUVECs treated with Exo-LVcircTUBGCP4 had more nodes than Exo-LVNC of HCT116 and SW480, then abolished by miR-146b mimic (Figs. 7h and S6a). Moreover, the laser confocal image displayed that there are more filopodia (yellow-marked) and higher CD34 expression in Exo-LVcircTUBGCP4-treated HUVECs than Exo-LVNC-treated HUVECs. However, the uptrend was arrested by miR-146b-3p (Figs. 7i-j and S6b). These results indicate that exosomal circTUBGCP4 enhances cell migration, tube formation, and tip cell formation of HUVECs by sponging miR-146b-3p.

## Discussion

**Fig. 8:**
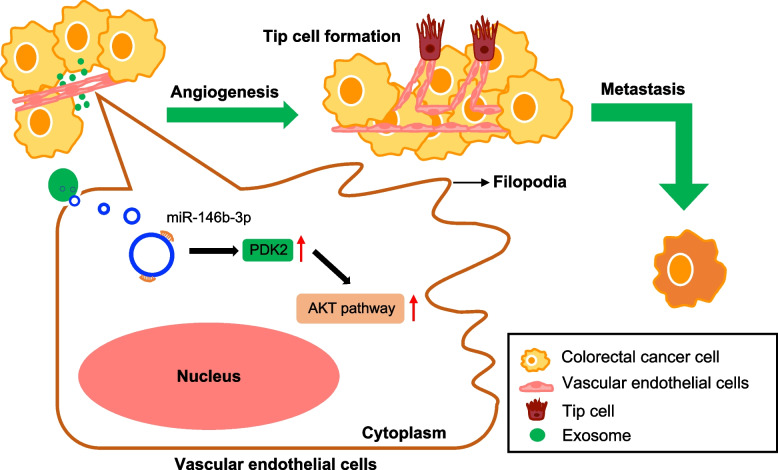
A schematic model of exosomal circTUBGCP4 function in angiogenesis and tumor metastasis

Here, we observed that exosome from cancer cells was quickly taken up by vascular endothelial cells and subsequently induced endothelial cell migration, tube formation, and tip cell migration of vascular endothelial cell. We further found that circRNAs were enriched in exosomes from cancer cells and serum from CRC patients with or without metastasis. In these differently expressed exo-circRNAs, the upregulated circTUBGCP4 was found to promote vascular endothelial cell migration, tube formation, and tip cell migration by targeting the miR-146b-3p to activate Akt signaling pathway (Fig. 8).

Growing evidence indicated that CRC-CDEs play an important role in cancer development and metastasis [5, 18]. Moreover, exosomes derived from various cell types play important roles in vascular development, growth, and maturation [19]. The CRC-CDEs were found to promote endothelial cell angiogenesis and break down vascular integrity, finally leading to tumor metastasis [20, 21]. It has been well recognized that the migration and tube formation of vascular endothelial cells is the essential step for angiogenesis. Similarly, our study shows that CRC-CDEs play an important role in the migration and tube formation of vascular endothelial cells. Moreover, our results which are not reported that CRC-CDEs can promote the formation of filopodia which are the main features of tip cells.

The competitive migration property of tip cells is the main process of sprouting angiogenesis, which is fundamental to development and contributes to cancer metastasis. Recently study reported the heterogeneity of lung tumor endothelial cells from patients, and mouse at the single-cell level. They found that NSCLC patients with squamous harbor high levels of tip cells, which predicted a poor prognosis [22]. It is well known that high VEGFs secreted by tumor cells can activate the VEGFR signaling pathway to maintain endothelial cell survival, proliferation, migration, and vascular proliferation, increase vascular permeability, and regulate tip cell formation [23]. Therefore, VEGF can be a target to inhibit tumor angiogenesis for tumor therapy. Bevacizumab is a commonly used VEGF-targeting drug as first-line therapy in metastatic colon cancer. However, its drug resistance has seriously affected the prognosis of CRC patients. Integrin β1 which is reported to promote endothelial sprouting is indispensable for vessel maturation [12]. Research showed that integrin β1 in maturing vessels is necessary for VE-cadherin localization and the integrity of cell–cell junction [12]. In our study, we found that integrinβ1 expression was upregulated after tumor cell exosome treatment. Moreover, high ITGB1 expression in CRC tissue predicted a poor prognosis. CD34 is an identified marker of tip cells in vascular endothelial monolayers in vitro [15]. A researcher found that CD34-positive endothelial cells demonstrated enrichment for biological functions related to angiogenesis and migration [15]. By analyzing the GEO database, we found that CD34 was slightly upregulated in the bevacizumab-resistant group compared to the non-resistant group. This result implied that there are other ways for tumor cells to induce tip cell formation to promote angiogenesis and tumor metastasis. In our study, we first reported that CRC cells can promote filopodia formation and tip cell formation by the delivery of exosomes. Our results found that CRC-CDEs could accumulate the expression of a tip cell marker, CD34, which represented an increased proportion of tip cells. High CD34 was correlated with CRC metastasis and predicted a bad prognosis.

Noncoding RNAs are the main contents of exosomes that contribute to tumor angiogenesis and metastasis [24, 25]. It has been reported that exosomal miR-25-3p from CRC cells promotes vascular leakiness, vascular permeability, and angiogenesis via targeting KLF2 and KLF4 to enhance tumor metastasis [26]. In addition, some lncRNAs, such as lncRNA H19, are enriched in exosomes released by CD90 + liver cancer cells. Moreover, the exosomal H19 could promote angiogenic phenotype and cell-to-cell adhesion of endothelial cells [27]. Many studies verify the essential role of exosomal miRNA and lncRNA in tumor angiogenesis and metastasis. However, the contribution of exosomal circRNAs regulating tumor angiogenesis to accelerate metastasis is still unknown. In our findings, we showed the enrichment, different types, and different expression profile of circRNAs in exosomes from CRC cells and serum of CRC patients with or without metastasis. Furthermore, we identified a potential CRC cell-released exosomal circTUBGCP4 that was upregulated in the serum of CRC patients with metastasis. Exosomal circTUBGCP4 showed a significant effect on promoting endothelial cell migration, tube formation, and the expression of CD34 Integrin β1 and VEGFA in HUVECs. Moreover, exosomal circTUBGCP4 could promote angiogenesis and tumor metastasis in vivo. Likewise, overexpressed circTUBGCP4 in CRC cells displayed the oncogene phenotypes of pro-angiogenic and tumor metastasis in vivo.

CircRNA has been reported to be involved in CRC progression and metastasis [28]. Our previous study reported that a YAP1-homologous circRNA, circ1662 promotes CRC metastasis by accelerating YAP1 nuclear localization [11]. Recently, increasing exosomal circRNAs were found to bind miRNA to contribute to cancer growth, metastasis, and drug resistance via binding miRNA or protein [29, 30]. Here, we found that circTUBGCP4 has potential binding sites to AGO2 protein, which combines with miRNA to package RNA-induced silencing complex (RISC) [31]. Then, we showed that circTUBGCP4 can bind AGO2 protein and be a sponge to target miR-146b-3p.

It has been early demonstrated that miRNA is a key modulator of angiogenic properties of human vascular endothelial cells [32]. A study revealed that miR-146b-3p could be a potential therapeutic intervention in preventing the dysfunction of microvascular associated with diabetic retinopathy (DR) [33]. In our study, we found that CRC-CDEs treatment led to a decrease in miR-146b-3p expression. Exogenous miR-146b-3p could inhibit endothelial cell migration, tube formation, and tip cell formation of HUVECs. Moreover, we found that miR-146b-3p is a key inhibitor to target PDK2 to suppress Akt signaling pathway, Previous findings showed that cancer cells increase endothelial cell tube formation and survival by activating the PI3K/Akt signaling pathway [17]. Activation of PI3K/Akt signaling pathway in endothelial cells promotes survival, migration, and tube formation [34]. Moreover, a network-Based analysis showed that PI3K/Akt signaling pathway and Rap1 signaling pathway in human vascular endothelial cells were the two pathways that differentiate into tip cells and stalk cells [35]. A report displayed that HtrA3 in HUVECs can enhance sprouting, cellular cortical protrusions, and mobility of HUVECs and further promote tip cell formation and tip position competition by activating PI3K/Akt signaling pathway [36]. In our study, we also found that CRC-CDEs and exosomal circTUBGCP4 can activate Akt signaling pathway to promote tip cell formation in vascular endothelial cells. And then, circTUBGCP4-sponged miR-146b-3p can target PDK2 and inhibit p-AKT to decrease CD34 expression. Our rescue results showed that circTUBGCP4 promotes the expression of PDK2, p-AKT, and CD34 depending on miR-146b-3p. Moreover, the role of circTUBGCP4 in migration, tube formation, and tip formation of vascular endothelial cells is regulated by miR-146b-3p. In the future, we will focus on the proposed mechanism of exosomal circTUBGCP4 in vivo to prove that exosomal circTUBGCP4 which promotes CRC angiogenesis and metastasis is dependent on miR-146b-3p. Moreover, we also need more clinical samples to analyze the correlation of exo-circTUBGCP4 with its targets and the expression of exo-circTUBGCP4, which will have a huge potential to be a biomarker for CRC therapy.

## Conclusion

In conclusion, our findings proved that CRC-CDEs induced more filopodia and vascular endothelial tip cell formation to promote cell migration and tube formation. Moreover, the circTUBGCP4 from CRC-CDEs was identified and can be transported into vascular endothelial cells to enhance cell migration, tube formation, and tip cell formation. Mechanically, we found circTUBGCP4 can target miR-146b-3p to trigger Akt signaling pathway in vascular endothelial cells. This study illuminates a new cancer-induced angiogenesis mechanism, which may provide a new approach to anti-angiogenesis therapy for CRC.

## Supplementary Information

**Additional file 1:** **Fig. S1.** The expression and prognosis of CD34 and ITGB1 in CRC samples from the GEO database. (a) High CD34 expression in the bevacizumab-resistant group compared to the bevacizumab-non-resistant group from GSE19860 and GSE19862 data. (b) High ITGB1 expression in the CRC group compared to the normal group from GSE71187. Then, the OS of ITGB1 in CRC was analyzed from the data of GSE71187. Log-rank test was used to estimate the significance. All data of GSE19860, 19862, and 71187 were reanalyzed using the BEST (https://rookieutopia.com/). **Fig. S2.** Silencing exosomal circTUBGCP4 inhibited the expression of CD34, integrin β1, VEGFA, and PDK2. (a) The CD34, integrin β1, and VEGFA expression of HUVECs treated with SW480-exosome derived from ShcircTUBGCP4-01 and ShcircTUBGCP4-02 stable cell lines were detected by Western blot. GAPDH was the internal control of whole-cell lysates. (b) The CD34 expression of HUVECs treated with HCT116-exosome derived from Sh-Circ stable cell lines were detected by immunofluorescence using confocal microscopy image. HUVECs incubated with PBS and ShNC-Exo were used as a negative control, Scale bars = 10 μm. (c) The PDK2 expression of HUVECs treated with exosome (HCT116 and SW480) derived from ShcircTUBGCP4-01 and ShcircTUBGCP4-02 stable cell lines were detected by Western blot. GAPDH was the internal control of whole-cell lysates. **Fig. S3. **Overexpressed circTUBGCP4 promoted migration and tube formation. (a) The overexpressed efficiency in HUVECs transfected circTUBGCP4 plasmid. (b) The migration and tube formation of HUVECs transfected overexpressed circTUBGCP4 plasmid were assessed using transwell migration assays and Tube formation assays. The number of migrated cells and nodes was analyzed by Image J and Prism 9; Mean ± SEM. Student's t-test, *P < 0.05, 0.001 < ** *P* < 0.01, *** *P* < 0.001. (c) The integrin β1 and VEGFA expression of HUVECs transfected overexpressed circTUBGCP4 plasmid were detected by Western blot. GAPDH was the internal control of whole-cell lysates. **Fig. S4**. The efficiency circTUBGCP4 overexpression and miR-146b-3p mimic in modified HUVEC. (a) The efficiency of circTUBGCP4 overexpression vector and miR-146b-3p mimic in modified HUVEC. (b) The efficiency of miR-146b-3p mimics in modified HUVECs. **Fig. S5.** The expression of miR-146b-3p in CRC samples from the TCGA database. (a) High expression of miR-146b-3p in the normal group compared with primary tumor from TCGA data. (b) Gradually low expression of miR-146b-3p in normal, stage 1, stage 2, stage 3, and stage 4 from TCGA data. (c) Gradually low expression of miR-146b-3p in normal, N0, N1, and N2 from TCGA data. **Fig. S6.** Exosomal circTUBGCP4 contribution to HUVECs dysfunction regulated by miR-146b-3p. (a) HUVECs were treated with exosomes derived from LVcircTUBGCP4-Exo (HCT116 and SW480) and its negative control, follow by transfected with miR-146b-3p mimic and NC mimic. After 48h, the migration and tube formation were measured through tube formation assay at 4h. The number of nodes was analyzed by Image J and Prism 9. (b) The CD34 immunofluorescence in the PBS+NC mimic, SW480-Exo-LVNC+ NC mimic, SW480-Exo-LVCirc+ NC mimic, SW480-Exo-L LVCirc+miR-146b-3p mimic, Scale bars = 10 μm. Mean ± SEM. Student's t-test, *P < 0.05, 0.001 < ** *P* < 0.01, *** *P* < 0.001. **Table S1.** The sequence of shRNA. **Table S2.** The sequence of primer (F: Forward primer; R: Reverse primer). **Table S3.** The sequence of probe. **Table S4.** The AGO2-binding sites of circTUBGCP4.

## Data Availability

All the data used in the current study are available from the corresponding authors upon reasonable request.

## Abbreviations

CRC-CDEs: Colorectal cancer cell-derived exosomes
HUVECs: Human umbilical vein endothelial cells
Exo-free FBS: Exosome-free fetal bovine serum
ISH: In-situ hybridization
IF: Immunofluorescence
IHC: Immunohistochemistry
RIP: RNA immunoprecipitation
OS: Overall survival
RFS: Relapse-free survival
DP: Divergent primer
CP: Convergent primer
AN: Adjacent normal tissue
LM: Liver metastasis
Non-LM: Non-liver metastasis
RISC: RNA-induced silencing complex
DR: Diabetic retinopathy
