# Supplementary material for: Exosomal circTUBGCP4 promotes vascular endothelial cell tipping and colorectal cancer metastasis by activating Akt signaling pathway
Source: J Exp Clin Cancer Res. 2023 Feb 15;42:46. doi: 10.1186/s13046-023-02619-y (PMC9930311; doi:10.1186/s13046-023-02619-y)
Supplement: Supplementary file 1 — Additional file 1: Fig. S1. The expression and prognosis of CD34 and ITGB1 in CRC samples from the GEO database. (a) High CD34 expression in the bevacizumab-resistant group compared to the bevacizumab-non-resistant group from GSE19860 and GSE19862 data. (b) High ITGB1 expression in the CRC group compared to the normal group from GSE71187. Then, the OS of ITGB1 in CRC was analyzed from the data of GSE71187. Log-rank test was used to estimate the significance. All data of GSE19860, 19862, and 71187 were reanalyzed using the BEST (https://rookieutopia.com/). Fig. S2. Silencing exosomal circTUBGCP4 inhibited the expression of CD34, integrin β1, VEGFA, and PDK2. (a) The CD34, integrin β1, and VEGFA expression of HUVECs treated with SW480-exosome derived from ShcircTUBGCP4-01 and ShcircTUBGCP4-02 stable cell lines were detected by Western blot. GAPDH was the internal control of whole-cell lysates. (b) The CD34 expression of HUVECs treated with HCT116-exosome derived from Sh-Circ stable cell lines were detected by immunofluorescence using confocal microscopy image. HUVECs incubated with PBS and ShNC-Exo were used as a negative control, Scale bars = 10 μm. (c) The PDK2 expression of HUVECs treated with exosome (HCT116 and SW480) derived from ShcircTUBGCP4-01 and ShcircTUBGCP4-02 stable cell lines were detected by Western blot. GAPDH was the internal control of whole-cell lysates. Fig. S3. Overexpressed circTUBGCP4 promoted migration and tube formation. (a) The overexpressed efficiency in HUVECs transfected circTUBGCP4 plasmid. (b) The migration and tube formation of HUVECs transfected overexpressed circTUBGCP4 plasmid were assessed using transwell migration assays and Tube formation assays. The number of migrated cells and nodes was analyzed by Image J and Prism 9; Mean ± SEM. Student's t-test, *P < 0.05, 0.001 < ** P < 0.01, *** P < 0.001. (c) The integrin β1 and VEGFA expression of HUVECs transfected overexpressed circTUBGCP4 plasmid were detected by Western blot. GAPDH w [file 13046_2023_2619_MOESM1_ESM.docx]

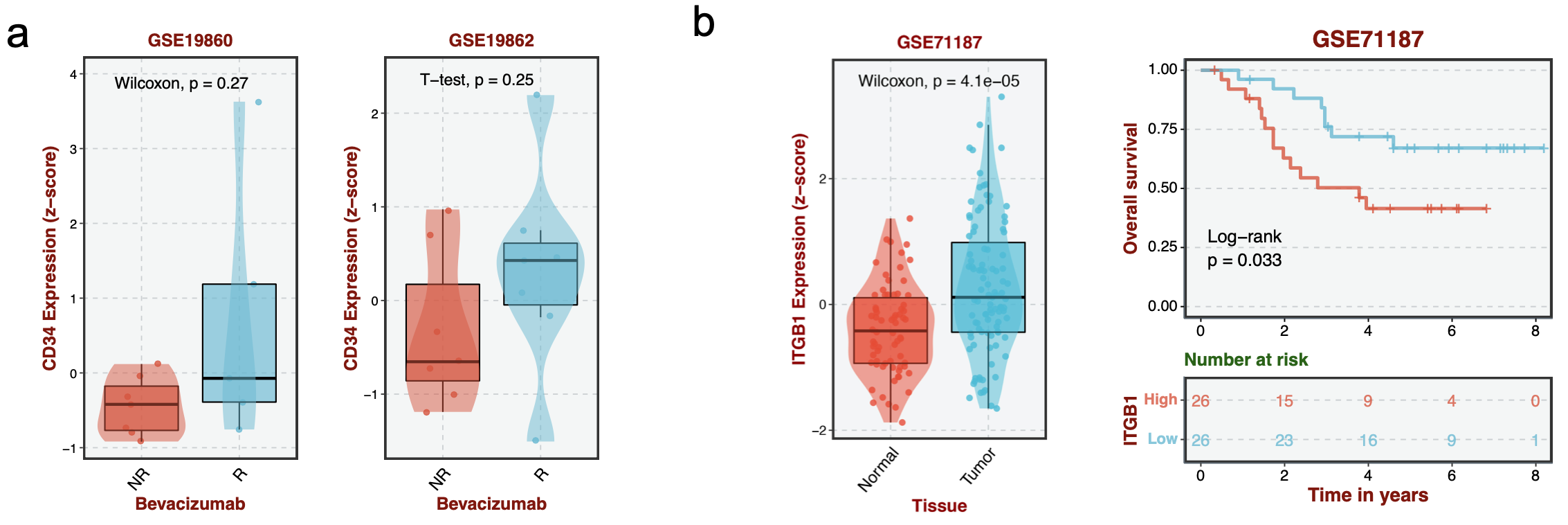


**Fig. S1 The expression and prognosis of CD34 and ITGB1 in CRC samples from the GEO database.**

(a) High CD34 expression in the bevacizumab-resistant group compared to the bevacizumab-non-resistant group from GSE19860 and GSE19862 data. (b) High ITGB1 expression in the CRC group compared to the normal group from GSE71187. Then, the OS of ITGB1 in CRC was analyzed from the data of GSE71187. Log-rank test was used to estimate the significance. All data of GSE19860, 19862, and 71187 were reanalyzed using the BEST (<https://rookieutopia.com/>).


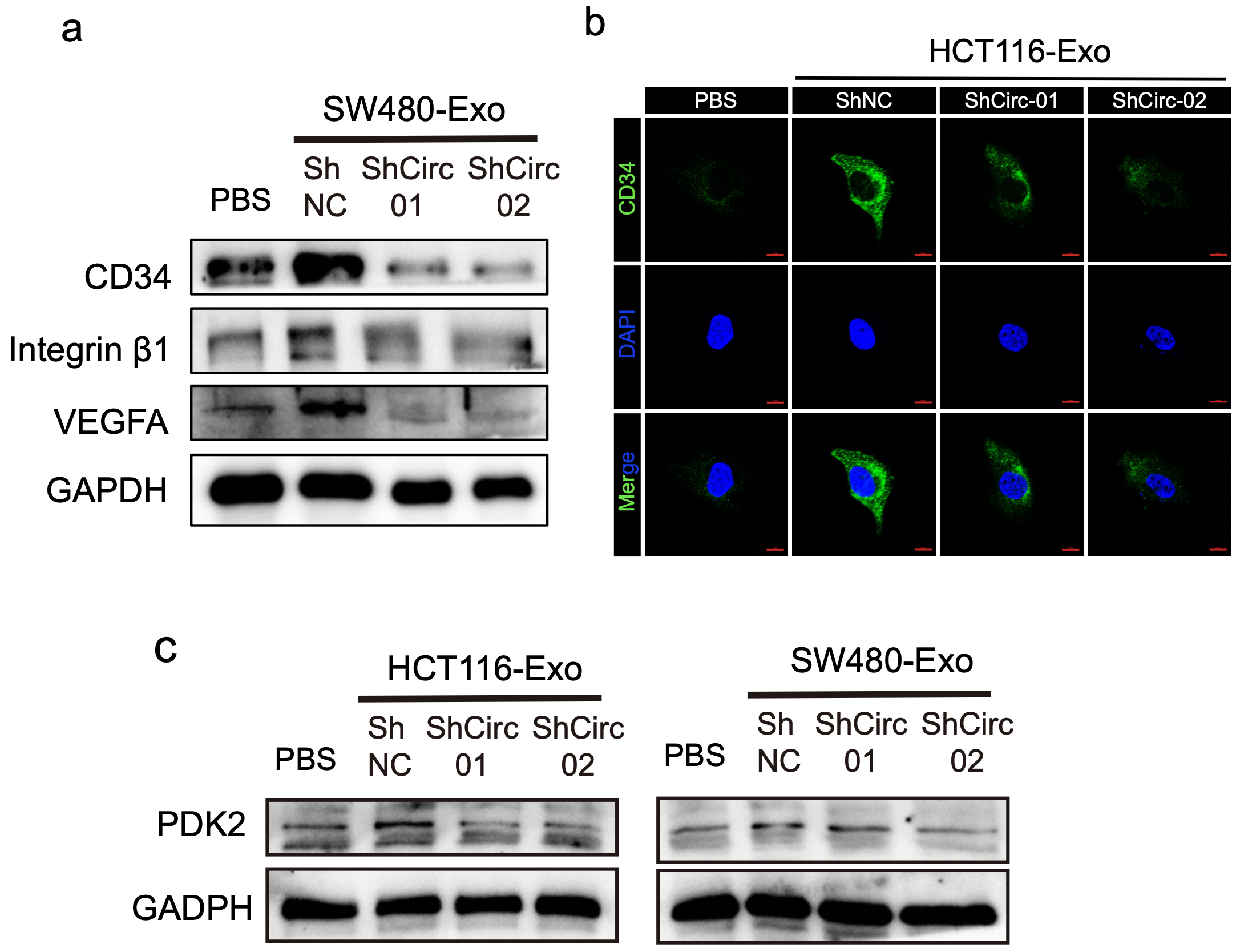


**Fig. S2 Silencing exosomal circTUBGCP4 inhibited the expression of CD34,** integrin β1, VEGFA, and PDK2**.**

(a) The CD34, integrin β1, and VEGFA expression of HUVECs treated with SW480-exosome derived from ShcircTUBGCP4-01 and ShcircTUBGCP4-02 stable cell lines were detected by Western blot. GAPDH was the internal control of whole-cell lysates. (b) The CD34 expression of HUVECs treated with HCT116-exosome derived from Sh-Circ stable cell lines were detected by immunofluorescence using confocal microscopy image. HUVECs incubated with PBS and ShNC-Exo were used as a negative control, Scale bars = 10 μm. (c) The PDK2 expression of HUVECs treated with exosome (HCT116 and SW480) derived from ShcircTUBGCP4-01 and ShcircTUBGCP4-02 stable cell lines were detected by Western blot. GAPDH was the internal control of whole-cell lysates.


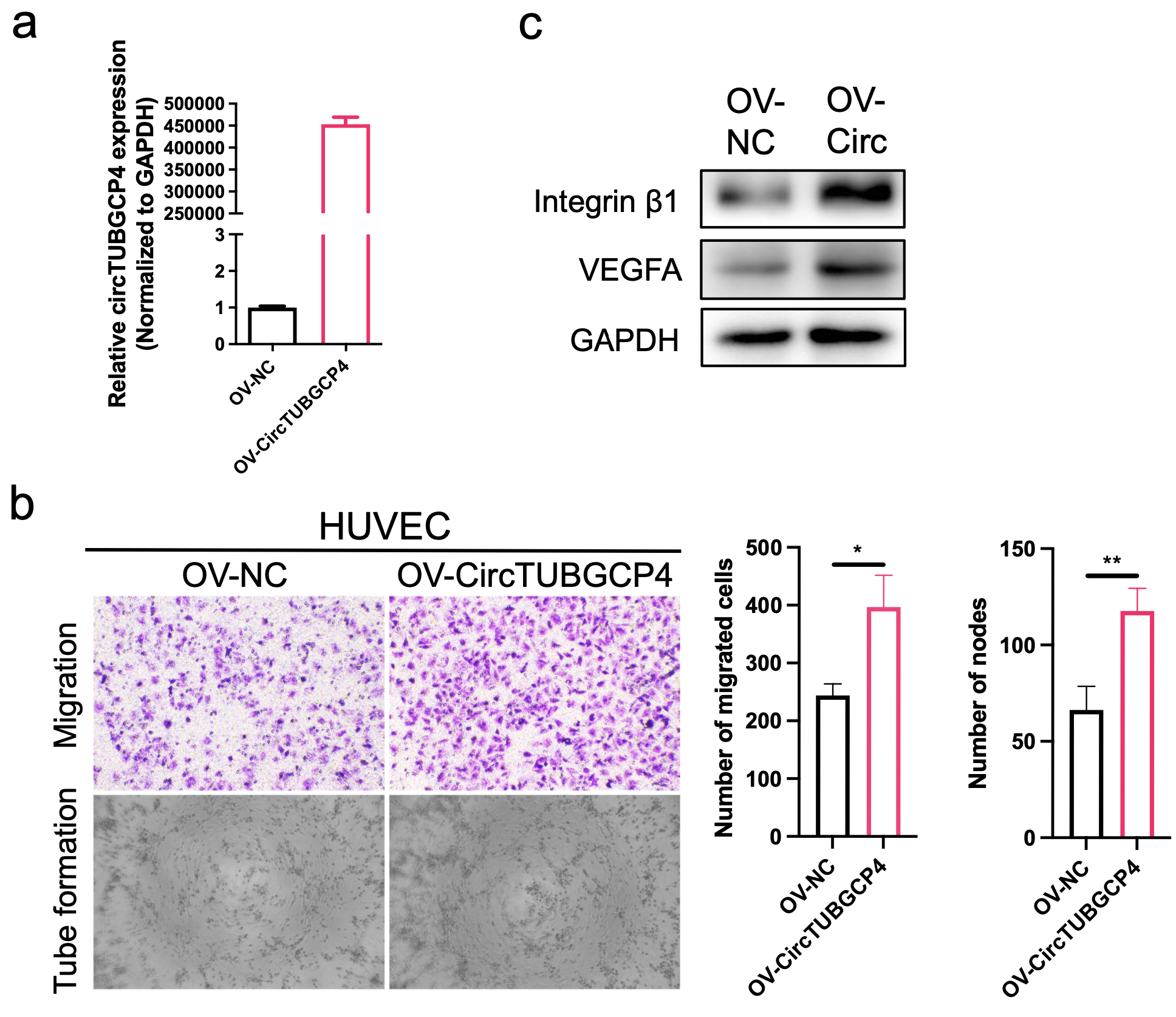


**Fig. S3 Overexpressed circTUBGCP4 promoted migration and tube formation**

(a) The overexpressed efficiency in HUVECs transfected circTUBGCP4 plasmid. (b) The migration and tube formation of HUVECs transfected overexpressed circTUBGCP4 plasmid were assessed using transwell migration assays and Tube formation assays. The number of migrated cells and nodes was analyzed by Image J and Prism 9; Mean ± SEM. Student's t-test, *P < 0.05, 0.001 < ** P < 0.01, *** P < 0.001. (c) The integrin β1 and VEGFA expression of HUVECs transfected overexpressed circTUBGCP4 plasmid were detected by Western blot. GAPDH was the internal control of whole-cell lysates.

**Fig. S4 The efficiency circTUBGCP4 overexpression and miR-146b-3p mimic in modified HUVEC.**

(a) The efficiency of circTUBGCP4 overexpression vector and miR-146b-3p mimic in modified HUVEC. (b) The efficiency of miR-146b-3p mimics in modified HUVECs.

**Fig. S5 The expression of miR-146b-3p in CRC samples from the TCGA database.**

(a) High expression of miR-146b-3p in the normal group compared with primary tumor from TCGA data. (b) Gradually low expression of miR-146b-3p in normal, stage 1, stage 2, stage 3, and stage 4 from TCGA data. (c) Gradually low expression of miR-146b-3p in normal, N0, N1, and N2 from TCGA data.


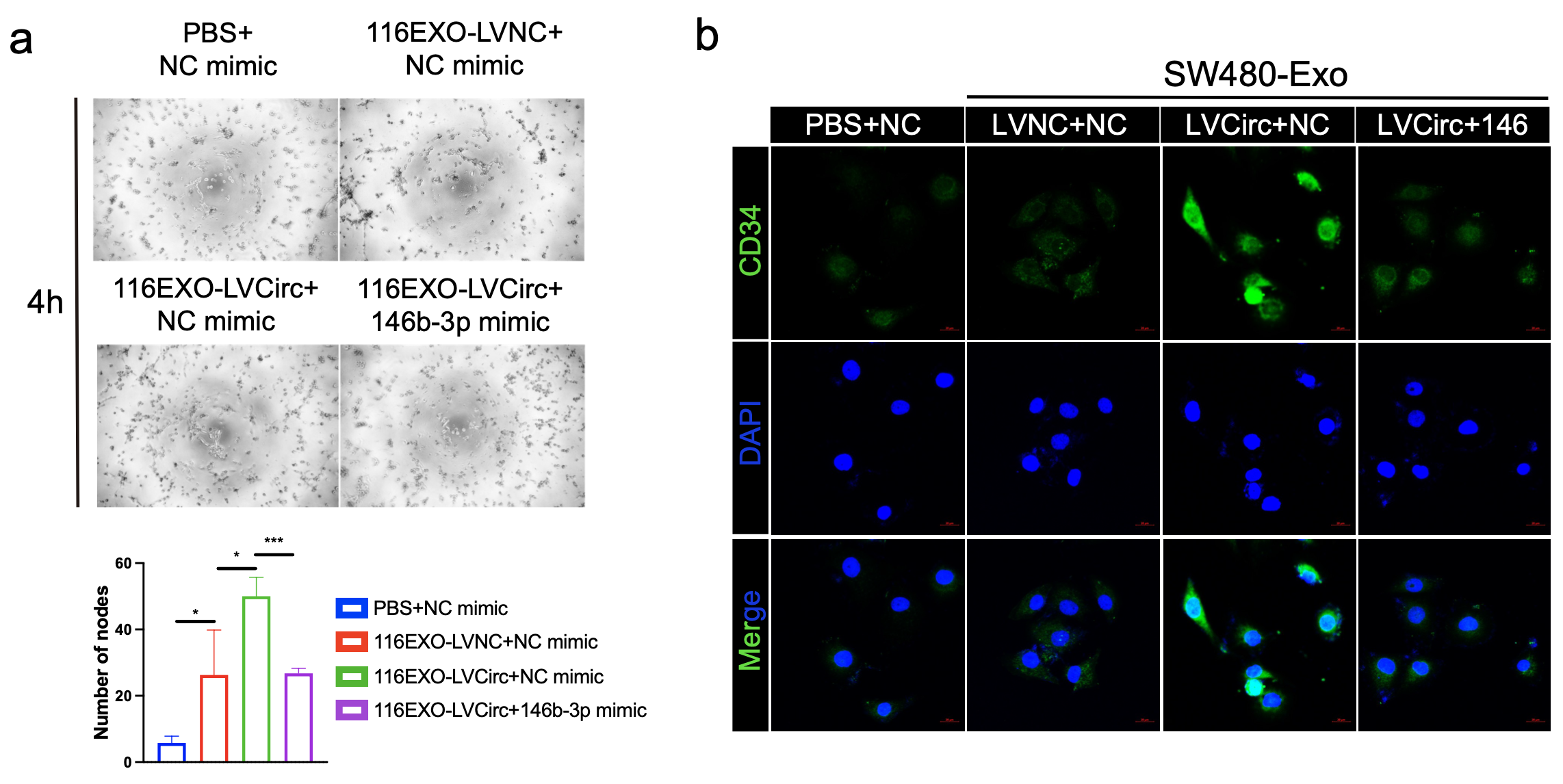


**Fig. S6 Exosomal circTUBGCP4 contribution to HUVECs dysfunction regulated by miR-146b-3p**

(a) HUVECs were treated with exosomes derived from LVcircTUBGCP4-Exo (HCT116 and SW480) and its negative control, follow by transfected with miR-146b-3p mimic and NC mimic. After 48h, the migration and tube formation were measured through tube formation assay at 4h. The number of nodes was analyzed by Image J and Prism 9. (b) The CD34 immunofluorescence in the PBS+NC mimic, SW480-Exo-LVNC+ NC mimic, SW480-Exo-LVCirc+ NC mimic, SW480-Exo-L LVCirc+miR-146b-3p mimic, Scale bars = 10 μm. Mean ± SEM. Student's t-test, *P < 0.05, 0.001 < ** P < 0.01, *** P < 0.001.

| **Table S1 The sequence of shRNA** | |
| --- | --- |
| Name | Target sequence(5'→3') |
| shcircTUBGCP4-01 | CCTAGACCAGGTATCGCAG |
| shcircTUBGCP4-02 | AGACCAGGTATCGCAGGAC |
|  |  |
| **Table S2 The sequence of primer (F: Forward primer;** **R: Reverse primer)** | |
| Name | Sequence(5'→3') |
| CircTUBGCP4-Divergent primer-F | TGGAACAAGAGTTCCTGGGTG |
| CircTUBGCP5-Divergent primer-R | ATGGCCCGTGTACTGTTCAA |
| CircTUBGCP4-Convergent primer-F | GCAGGACTTCCCTTTCCTCC |
| CircTUBGCP4-Convergent primer-R | ACCTTGGCCCTGTTGAGATG |
| TUBGCP4-F | GGTATCGCAGGACTTCCCTTT |
| TUBGCP4-R | TGGCCCGTGTACTGTTCAATG |
| PDK2-F | ATGAAAGAGATCAACCTGCTTCC |
| PDK2-R | GGCTCTGGACATACCAGCTC |
| GAPDH-F | AACGGATTTGGTCGTATTGG |
| GAPDH-R | TTGATTTTGGAGGGATCTCG |
| miR-146b-3p (ploy A) | Ribo-miRA1000403 |
| **Table S3 The sequence of probe** | |
| Name | probe (5'→3') |
| CircTUBGCP4-ISH probe | 5'DIG-GGAAGTCCTGCGATACCTGGTCTAGGAAGTAGTT |
| CircTUBGCP4-biotin probe | 5'Biotin-CGA+TACCTGGTCTAGGAAGTAGT+TG |
| miR-146b-3p-biotin probe | 5'Biotin-GCCCTGTGGACTCAGTTCTGGT |
|  |  |

| **Table S4 The AGO2-binding sites of circTUBGCP4** | | | |
| --- | --- | --- | --- |
| RBP | Start | End | Details |
| AGO2_Human_GSE42701_HITS-CLIP | 43668374 | 43668427 | HHFKP_49987_cluster-7262_2_154 |
| AGO2_Human_GSE28865_HITS-CLIP | 43668381 | 43668421 | HHKTA_6302_NM_014444\|405\|444\|10.0814302650594\|0.617769715000406 |
| AGO2_Human_GSE28865_PAR-CLIP | 43668392 | 43668421 | HPKMA_4864_G5680.1_43668409 |
| AGO2_Human_GSE32109_PAR-CLIP | 43668394 | 43668421 | HPCB3_7538_G10374.1_43668409 |
| AGO2_Human_GSE32109_PAR-CLIP | 43668395 | 43668421 | HPCB1_13697_G17260.1_43668409 |
| AGO2_Human_GSE41437_PAR-CLIP | 43668400 | 43668421 | HPSEF_7733_G9288.1_43668409 |
| AGO2_Human_GSE21918_PAR-CLIP | 43668400 | 43668421 | HPTK1_10370_G12305.1_43668409 |
| AGO2_Human_GSE21918_PAR-CLIP | 43668400 | 43668420 | HPTK7_2638_G3854.1_43668409 |
| AGO2_Human_GSE43573_PAR-CLIP | 43668400 | 43668420 | HPRT3_8963_G11794.1_43668409 |
| AGO2_Human_GSE41437_PAR-CLIP | 43668400 | 43668421 | HPSLC_6685_G7911.1_43668409 |
| AGO2_Human_GSE28865_PAR-CLIP | 43668400 | 43668421 | HPKTB_3682_G4654.1_43668409 |
| AGO2_Human_GSE28865_PAR-CLIP | 43668400 | 43668421 | HPKTA_20826_G22442.1_43668409 |
| AGO2_Human_GSE42701_HITS-CLIP | 43668550 | 43668582 | HHFKP_49988_cluster-7262_3_8 |
| AGO2_Human_GSE28865_PAR-CLIP | 43668741 | 43668767 | HPKMA_4865_G5681.1_43668767 |
